# Spindle-Shaped Neurons in the Human Posteromedial (Precuneus) Cortex

**DOI:** 10.3389/fnsyn.2021.769228

**Published:** 2022-01-11

**Authors:** Francisco Javier Fuentealba-Villarroel, Josué Renner, Arlete Hilbig, Oliver J. Bruton, Alberto A. Rasia-Filho

**Affiliations:** ^1^Department of Basic Sciences/Physiology, Universidade Federal de Ciências da Saúde de Porto Alegre, Porto Alegre, Brazil; ^2^Graduate Program in Neuroscience, Universidade Federal do Rio Grande do Sul, Porto Alegre, Brazil; ^3^Graduate Program in Biosciences, Universidade Federal de Ciências da Saúde de Porto Alegre, Porto Alegre, Brazil; ^4^Department of Medical Clinics/Neurology, Universidade Federal de Ciências da Saúde de Porto Alegre, Porto Alegre, Brazil; ^5^Carl von Ossietzky Universität Oldenburg, Oldenburg, Germany

**Keywords:** human brain (cerebral cortex), cytology (CY), default mode network (DMN), dendritic spines, 3D reconstruction, general intelligence (*g*), von Economo neuron (VEN)

## Abstract

The human posteromedial cortex (PMC), which includes the precuneus (PC), represents a multimodal brain area implicated in emotion, conscious awareness, spatial cognition, and social behavior. Here, we describe the presence of Nissl-stained elongated spindle-shaped neurons (suggestive of von Economo neurons, VENs) in the cortical layer V of the anterior and central PC of adult humans. The adapted “single-section” Golgi method for *postmortem* tissue was used to study these neurons close to pyramidal ones in layer V until merging with layer VI polymorphic cells. From three-dimensional (3D) reconstructed images, we describe the cell body, two main longitudinally oriented ascending and descending dendrites as well as the occurrence of spines from proximal to distal segments. The primary dendritic shafts give rise to thin collateral branches with a radial orientation, and pleomorphic spines were observed with a sparse to moderate density along the dendritic length. Other spindle-shaped cells were observed with straight dendritic shafts and rare branches or with an axon emerging from the soma. We discuss the morphology of these cells and those considered VENs in cortical areas forming integrated brain networks for higher-order activities. The presence of spindle-shaped neurons and the current discussion on the morphology of putative VENs address the need for an in-depth neurochemical and transcriptomic characterization of the PC cytoarchitecture. These findings would include these spindle-shaped cells in the synaptic and information processing by the default mode network and for general intelligence in healthy individuals and in neuropsychiatric disorders involving the PC in the context of the PMC functioning.

## Introduction

Von Economo neurons (VENs) have a peculiar phylogenetic and ontogenetic development and are characterized by an elongated spindle-shaped or rod-shaped cell body mainly found in the cortical layer V of restricted brain areas of some species, including humans and other primates, but not in all mammals (Nimchinsky et al., 1995; Allman et al., 2005, 2010; Hakeem et al., 2009; Cauda et al., 2014; Raghanti et al., 2015; Hodge et al., 2020; Jacob et al., 2021). In humans, VENs have been reported mainly in the anterior cingulate cortex (ACC) and frontoinsular cortex (FI; Raghanti et al., 2015; Banovac et al., 2019, 2021; Correa-Júnior et al., 2020 and references therein), but also in the dorsolateral prefrontal cortex (Brodmann area 9; Fajardo et al., 2008) and in the medial frontopolar prefrontal cortex (Brodmann area 10; González-Acosta et al., 2018). VENs might correspond to ∼3% of all neurons in layer V in the ACC (Fajardo et al., 2008; see comments about their relative abundance in Banovac et al., 2021), being more numerous in the ACC and FI of humans than in apes (Allman et al., 2010).

Human VENs show features of excitatory projecting neurons (Nimchinsky et al., 1995; Evrard et al., 2012; Hodge et al., 2020). They may provide fast interconnections between neocortical areas, such as the ACC and FI cortices (Craig, 2009), and recent transcriptomic data suggest that human VENs may project to extratelencephalic, subcortical targets (Hodge et al., 2020). In gorillas, VENs also project to the inferior frontal gyrus, inferotemporal cortex, laterally to the hippocampus, as well as to the septum and amygdala (Allman et al., 2010). VENs innervate the midbrain periaqueductal gray and the parabrachial nucleus of dorsolateral pons of monkeys (discussed in Evrard et al., 2012), and may link emotion and control of sympathetic/parasympathetic sites in brainstem and spinal cord regions (Cobos and Seeley, 2015; Jacot-Descombes et al., 2020).

The identification of cells as VENs (or putative ones) has been based on Nissl/thionin staining (Nimchinsky et al., 1995; Raghanti et al., 2015), Golgi impregnation (Watson et al., 2006; Banovac et al., 2019, 2021; Correa-Júnior et al., 2020), intracellular biocytin (Hodge et al., 2020), retrograde labeling with cholera toxin b and Alexa 594 fluorescent dextran nanoinjection (Evrard et al., 2012), immunoreactivity for different biochemical biomarkers including neurotransmitters’ receptors, neuropeptides, and transcription factors (Allman et al., 2005, 2010; Stimpson et al., 2011; Dijkstra et al., 2018), and/or different gene expressions (Allman et al., 2010; Evrard et al., 2012; Cobos and Seeley, 2015; Yang et al., 2019; Hodge et al., 2020). VENs are morphologically different from neighboring pyramidal neurons (Allman et al., 2005; Watson et al., 2006) and are larger than layer VI “fusiform” neurons (Nimchinsky et al., 1999). Although having some molecular profile in common with layer V pyramidal and fork neurons, human putative VENs showed distinctive intrinsic membrane properties in the FI (Hodge et al., 2020) and transcriptomic characteristics in the ACC (Yang et al., 2019). These data are fundamental to integrate morphological and genetic characteristics to identify phenotype-associated cells and to test the proposed functional roles for VENs in main control neural networks (Bruton, 2021), as well as their vulnerability in diseases with social and emotional deficits (Allman et al., 2005; Cauda et al., 2014; Raghanti et al., 2015).

The shape of VENs is also characterized by the presence of two main perpendicularly oriented thick primary dendritic shafts, one ascending toward more superficial cortical layers and another descending toward the inner cortical layer (Allman et al., 2005; Watson et al., 2006; Evrard et al., 2012; Seeley et al., 2012; Raghanti et al., 2015). Indeed, the human ACC is a region where the cell body and the primary dendrites of VENs were identified using different morphological, neurochemical, and transcriptomic approaches. Based on these data to guide the use of the Golgi method, the three-dimensional (3D) reconstruction of Golgi-impregnated layer V VENs indicated the heterogeneity of dendrites and spines of these cells in this brain area. That is, ACC VENs were described in a morphological *continuum* from sparsely branched (as previously reported by Watson et al., 2006) to more extensively ramified cells with varied collateral branches and differences in the distribution, density, and shape of dendritic spines (Correa-Júnior et al., 2020). On the other hand, VENs should have a brush-like aspect for the descending dendrites with an axon in the ACC and in the FI, a morphological characteristic that was evidenced in the first drawings of these Golgi-impregnated neurons (Banovac et al., 2019, 2021; note the aspect of the axonal ramification in the original drawing from Ramón y Cajal shown as Figure 1 in Banovac et al., 2021). Dendrites and spines in VENs are important cellular elements to be studied because the geometry and biophysical properties of both are linked to the synaptic integration, strength, and plasticity (Yuste, 2010; Rochefort and Konnerth, 2012; Spruston et al., 2013; Hayashi-Takagi et al., 2015; Rollenhagen and Lübke, 2016; Tønnesen and Nägerl, 2016; Nakahata and Yasuda, 2018) for broad information processing in healthy individuals and in neurodegenerative diseases (Herms and Dorostkar, 2016). Dendritic spines are complex elements that can enhance the connectivity between neurons and increase the packing density of synapses without increasing the overall volume of the brain (Bourne and Harris, 2009). Moreover, dendritic spines are specialized postsynaptic units for most excitatory inputs (Spruston et al., 2013; Brusco et al., 2014; Helm et al., 2021; but see Kubota et al., 2016 for the impact of inhibitory GABAergic terminals on spines), whose complexity is evident in the human brain (Cajal, 1909-1911; Yuste, 2013; Dall’Oglio et al., 2015).

The neuroanatomical and cytoarchitectonic maps of the posteromedial cortex (PMC), including the subdivisions of the precuneus (PC, former “quadrate lobule of Foville”), represent current research avenues on multimodal integrative areas in the human brain. The anatomical and functional development of the PC as well as the adjacent medioventral areas in the PMC may have played a significant role in human brain evolution for somatosensory processing, motor behavior, mental imagery, attentional shift, self-awareness, and judgments about other persons’ mental states, social and cognitive specializations (Wenderoth et al., 2005; Denny et al., 2012; Bruner et al., 2014, 2017a,b). The human PMC may include the PC, the posterior cingulate cortex (Brodmann area, BA, 23), the retrosplenial cortex in the posterior callosal sulcus (areas 29 and 30), and the transitional zone (area 31) between the PC and the posterior cingulate cortex (Margulies et al., 2009). Some features are remarkable: (1) bulging of the parietal surface during the first year of life in *Homo sapiens* is a morphogenetic stage absent in chimpanzees and Neandertals, (2) the deep parietal areas show discrete cytoarchitectural differences between human and non-human primates, and (3) the extension of the PC is the principal source of midsagittal brain variability in adult humans (Bruner et al., 2014, 2017a,b and references therein). In midsagittal sections, the PC is a fully differentiated isocortex located in the superior parietal cortex, posterior to the postcentral sulcus and the marginal ramus of the cingulate sulcus, anterior to the parieto-occipital fissure and the cuneus, above the subparietal sulcus and adjacent to the transition area to the posterior cingulate and the retrosplenial cortex (Cavanna and Trimble, 2006; Scheperjans et al., 2008a,b; Margulies et al., 2009; Mai et al., 2016; Bruner et al., 2017a). Nevertheless, the human PC varies in its geometry (mostly in its longitudinal dimensions), in the patterns of convolution, and how sulci extend within it (Bruner et al., 2014, 2017a).

The human PC has been referred to as BA 7 (or 7m, further divided in 7a and 7b for its anterior and posterior parts, respectively) or, according to the von Economo and Koskinas cytoarchitectonic atlas, numbered as areas 62–64 (or P*Em*, P*Ep*, and P*Ey*; c.f. Triarhou, 2009). The human PC would include BAs 7 and 31 or, additionally, the 23 and 30 ones (e.g., see plates 72 and 77 in Mai et al., 2016; but see data in Cavanna and Trimble, 2006 and references therein). Otherwise, the cytoarchitectonic parcellation of structurally distinct microanatomical areas identified a heterogeneous medial border for the 7A, 7P, and 7M parts in the human PC, considering the existence of interindividual anatomical variability and interhemispheric topographic asymmetries (Scheperjans et al., 2008a,b). These PC parts would be located posterior to the postcentral sulcus, where areas 5L and 5M would still be occupying a variable volume of the anterior part of the Brodmann PC 7a (Scheperjans et al., 2008a,b). Functionally, the PC is a multimodal integrative heterogeneous area as revealed by resting-state functional MRI data (Margulies et al., 2009). That is, (1) the anterior PC can be a sensorimotor region connected with the superior parietal cortex, paracentral lobule, and motor cortex, also including the insula; (2) the central PC can be a cognitive/associative/multimodal zone connected to the dorsolateral prefrontal, dorsomedial prefrontal, and multimodal lateral inferior parietal cortex; and (3) the posterior PC is connected with contiguous visual cortical regions (Margulies et al., 2009).

Considering that VENs occur in cortical areas for higher sensorial and motor integration, emotional and cognitive functions, intuition and social behavior elaboration, and flexibility, we looked for the presence of “spindle-shaped” cells, which would be suggestive of VENs, in the human PMC. Our intention is not to cause a potential confusion with other uses of the term “spindle neurons” (also mentioned in Watson et al., 2006) or “spindle-transformed pyramidal cells” (as extensively reviewed in Banovac et al., 2021). Rather, we describe the morphology of these neurons in layer V until merging with layer VI of the human PC based on their Nissl-stained and Golgi-impregnated features while we do not have complete data for their definitive neurochemical and transcriptomic characterization and classification. Here, we studied the anterior and central regions of the PC (BA 7), which correspond approximately to fMRI chosen regions of interest/“seeds” 5–7 and 9–11 within the PC (Margulies et al., 2009; seeds data 11 and 15 from *SI Appendix* were visually compared and considered as central PC). Our samples included the tissue posterior to the postcentral sulcus, mostly corresponding to the PC (although areas 5L and 5M would also be included; Scheperjans et al., 2008a,b), and a small part of the transitional area 31 in the ventral limit close to the subparietal sulcus (but not advancing to the posterior cingulate cortex). First, we used Nissl staining to identify neurons with a characteristic elongated spindle-shaped cell body close to pyramidal neurons in the PC cortical layer V and in the transition to layer VI. Afterward, two-dimensional (2D) and 3D reconstructions of Golgi-impregnated spindle-shapled neurons evidenced two main shafts oriented perpendicularly, corresponding to the ascending and descending primary dendrites in these cells. We discuss the morphology of putative VENs in other human brain areas, the need for additional classification of these PC spindle-shaped cells as well as the implications for the local cytoarchitectonics and the functional possibilities for the presence of spindle-shaped (or putative VENs) in the human PC for both the “default mode network” (DMN) and high multimodal cortical processing.

## Materials and Methods

The present procedure was adapted from the description published in Correa-Júnior et al. (2020).

### Subjects

The subjects were two men and one woman reportedly healthy neurologically and psychiatrically, with no previous neurosurgical interventions. Age, sex, *postmortem* interval, and cause of death are shown in Table 1. The next of kin provided written informed consent for brain donation during an autopsy at the morgue, as well as provided the donors’ clinical and comorbidities information. Each subject was screened for cognitive decline using the “Informant Questionnaire on Cognitive Decline in the Elderly” (IQCODE; Neto et al., 2017). This validated interview procedure has a cut-off point scores indicative of dementia of ≥3.27 or 3.48 in the Brazilian population (Sanchez and Lourenço, 2009; Carrabba et al., 2015). Only cases below these values were included in the present study (Table 1). Brain tissue was also analyzed histologically and immunohistochemically by a neurologist/neuropathologist (AH) to confirm the absence of common vascular lesions or evident neurodegenerative disorders other than primary age-related alterations.

**TABLE 1:** Characteristics of the human cases.

Cases (code#)	Age (years)	Sex	PMI (hours)	IQCODE	Cause of death	Tissue fixation	Technique
2	91	F	6–20	1.32	Pneumonia	Immersion	Thionin/Golgi
3	79	M	6–20	3.15	Cardiac Arrest	Immersion	Thionin/Golgi
4	49	M	6–20	3.00	Undetermined	Immersion	Thionin/Golgi

*PMI, postmortem interval; F, female; M, male.*

All ethical and legal procedures were carried out in accordance with the international regulatory standards based on the Helsinki Declaration of 1964. The privacy rights of subjects were observed at all times. There is no potentially identifiable data for any individual included in this article. The Brazilian Ethics Committee from the Federal University of Health Sciences of Porto Alegre (UFCSPA; #62336116.6.0000.5345, 18718719.7.0000.5345, and 06273619.7.0000.5345) and the Federal University of Rio Grande do Sul (#18718719.7.3001.5347) approved this study.

### Tissue Processing and the Nissl Staining Procedure

**FIGURE 1:**
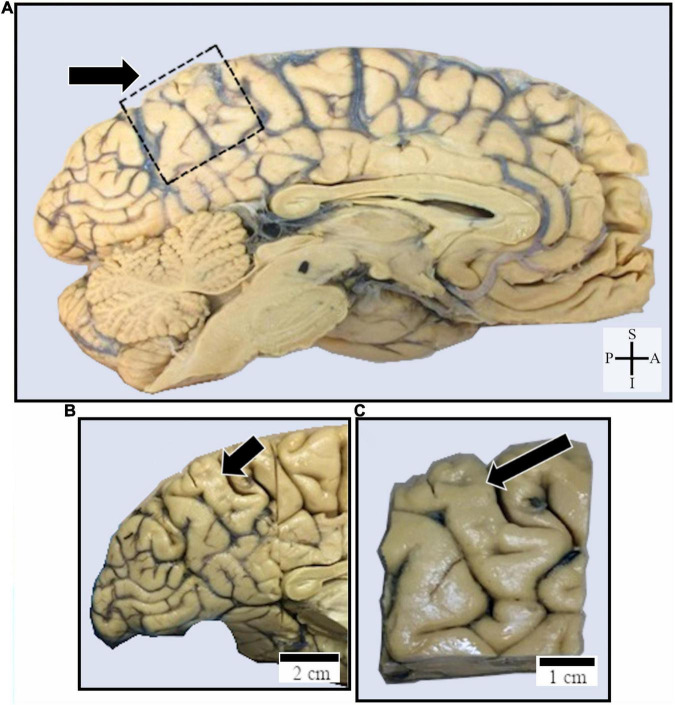
**(A)** Midsagittal section to show the macroscopic location of the human posteromedial cortex where the precuneus (PC, pointed by an arrow and delimited within a dashed square) was studied near the transition zone to the posterior cingulate cortex. **(B,C)** Higher magnification images of the human PC where samples were obtained for the identification of spindle-shaped neurons in cortical layer V (shown in the following figures). Coordinates: A, anterior; I, inferior; P, posterior; S, superior. Scales are shown in the figure.

**FIGURE 2:**
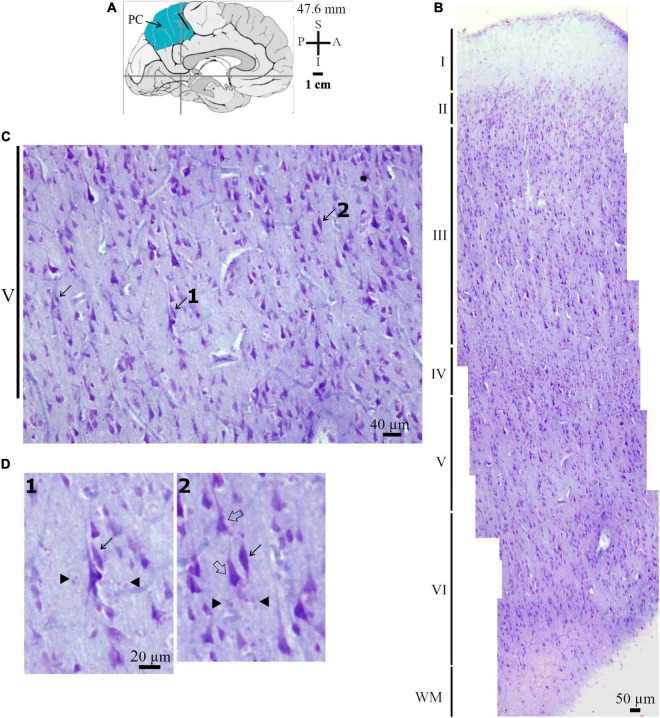
**(A)** Schematic drawing of the medial view of the human brain and the location of the anterior region of the precuneus (PC, highlighted in blue), in this case 47.6 mm posterior to the midpoint of the anterior commissure. The dark bar indicates approximately the place where histological data were obtained. Adapted from Mai et al. (2008, 2016). Coordinates: I, inferior; L, lateral; M, medial; S, superior. **(B)** Photomicrograph of Nissl-stained cells in layers I to VI in the anterior PC. WM, white matter. **(C)** Photomicrograph in higher magnification of Nissl-stained cells in the layer V of the human PC as shown in **(B)**. The spindle-shaped neurons (pointed by solid arrows) are close to pyramidal neurons and other local cells in the cortical layer V and in the transition to layer VI. **(D)** Further details for the morphology of the spindle-shaped neurons pointed and numbered 1 and 2 in **(C)**. Note their characteristic elongated cell body and two perpendicularly oriented primary dendrites, one with an ascending and another with a descending spatial orientation in the neuropil. Compare these spindle-shaped cells to the neighboring stained pyramidal neurons (examples indicated by an open arrow). Adjacent glia cells are also present (indicated by a solid arrow head). Scales are shown in each figure. Image adjustment of contrast and brightness made with Photoshop CS3 (Adobe Systems, Inc., United States).

**FIGURE 3:**
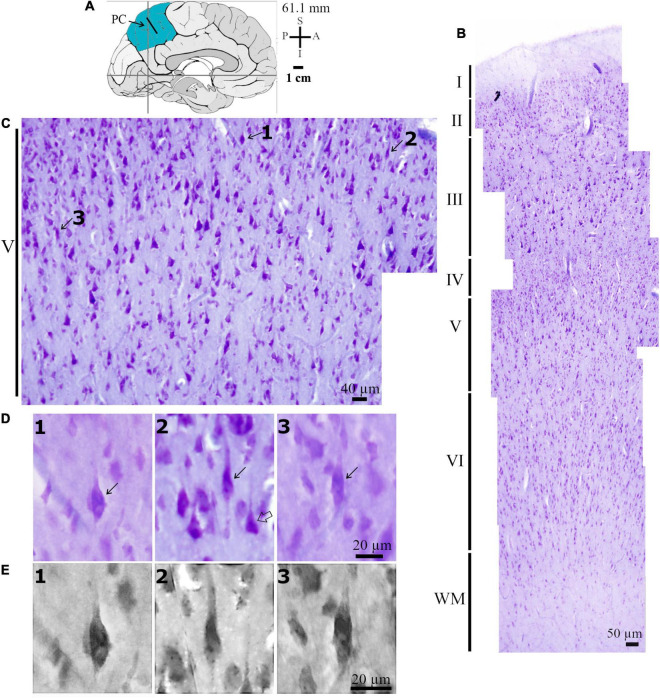
**(A)** Left: Schematic drawing of the medial view of the human brain and the location of the central region of the precuneus (PC, highlighted in blue), in this case 61.1 mm posterior to the midpoint of the anterior commissure. The dark bar indicates approximately the place where histological data were obtained. Adapted from Mai et al. (2008, 2016). Coordinates: I, inferior; L, lateral; M, medial; S, superior. **(B)** Photomicrograph of Nissl-stained cells in layers I to VI in the intermediate PC. WM, white matter. **(C)** Photomicrograph in higher magnification of Nissl-stained cells in the layer V of the human PC as shown in **(B)**. The spindle-shaped neurons (numbered 1–3 and pointed by solid arrows) are close to pyramidal neurons and other local cells in the cortical layer V and in the transition to layer VI. **(D)** Further detail for the morphology of these spindle-shaped neurons indicated in **(C)**. Note their characteristic elongated cell body and two perpendicularly oriented primary dendrites, one with an ascending and another with a descending spatial orientation in the neuropil. Compare these spindle-shaped cells to the neighboring stained pyramidal neurons (example indicated by open arrow). **(E)** Higher magnification of the same spindle-shaped neurons shown in **(D)** to evidence the aspect of their chromatin and nucleolus. Scales are shown in each figure. Image adjustment of contrast and brightness made with Photoshop CS3 (Adobe Systems, Inc., United States).

Brains were kept immersed in 10% laboratory-grade, unbuffered formaldehyde solution at room temperature (RT) for approximately 6 years before study. The PC was located in the dorsal portion of the PMC, posterior to the marginal ramus of the cingulate sulcus, anterior to the parieto-occipital fissure and the cuneus, and including the subparietal sulcus as anatomical reference, as mentioned above (Figure 1; Margulies et al., 2009; Mai et al., 2016; Bruner et al., 2017a). The anterior and central PC in the left hemisphere (the side available in these samples) were studied from 41.5 to 73.6 mm posterior to the midpoint of the anterior commissure (Mai et al., 2008, 2016; Figures 2, 3).

At the beginning of this study, tissue blocks were sectioned and post-fixed at RT for 30 days using phosphate buffer solution (PBS, 0.1 M, pH = 7.4), 4% formaldehyde, and 1.5% picric acid. Samples were coronally sectioned with a vibrating microtome (1000S; Leica, Germany) in an alternating fashion. One series was sectioned at 50 μm for the Nissl technique, the other at 200 μm for the Golgi method.

The Nissl staining identified the cortical layers and different cells in the PC (Figures 2, 3). That is, sections were (1) placed on gelatin-coated slides and left to dry at RT for 1 day; (2) then, slides were immersed in a 4% formaldehyde in PBS for 1 week at 4°C and protected from light; (3) dried for 1 day at RT and immersed in a 70% ethanol solution for 1 day; (4) immersed in increasing concentrations of ethanol and cleared in absolute xylene; (5) immersed in decreasing solutions of ethanol and washed in distilled water; (7) immersed in a solution of 0.25% cresyl violet (Merck, Germany) for 2 min; (7) immersed in distilled water to remove excessive dying, and in solutions of increasing ethanol concentration; (8) dipped in a solution of 95% ethanol with 1% acetic acid and absolute xylene; and (9) mounted with synthetic balsam (Soldan, Brazil) and coverslipped.

### The Golgi Method and the Two-Dimensional and Three-Dimensional Image Reconstruction Procedures

We used the “single-section” Golgi method adapted for long-term fixed *postmortem* human brain (Dall’Oglio et al., 2010 on the original Gabbott and Somogyi, 1984; developed for 3D image processing by Reberger et al., 2018) as previously done for the characterization of neurons and dendritic spines in both subcortical (amygdaloid nuclei) and cortical human brain areas (Dall’Oglio et al., 2013, 2015; Vásquez et al., 2018; Correa-Júnior et al., 2020; Rasia-Filho et al., 2021). Brain sections were sectioned and kept immersed in the same post-fixation solution as mentioned above for three more days. Afterward, sections were: (1) rinsed in PBS and immersed in a solution of 0.1% osmium tetroxide (Sigma Chemicals Co., United States) in PBS for 20 min; (2) rinsed in PBS and immersed in 3% potassium dichromate (Merck) at 4°C in the dark for 2 days; (3) rinsed in distilled water, “sandwiched” between coverslips, placed in a solution of 1.5% silver nitrate (Merck) at RT for 1 day and protected from light; (4) washed in distilled water, placed on gelatin-coated histological slides, dried at RT, dehydrated in an ascending series of ethanol, cleared in ethanol and absolute xylene; and (5) covered with non-acidic synthetic balsam (refractive index = 1.518–1.521, Permount Mounting Medium, EMS, United States or similar product, Soldan, Brazil) and coverslipped.

We used the following criteria to select Golgi-impregnated neurons for further study: (1) cells should have their soma located within the boundaries of the PC; (2) spindle-shaped cells should be near to pyramidal neurons in the cortical layer V until the transition to layer VI; (3) cells should have two main primary dendrites oriented vertically toward the white matter and apical surface; (4) cells should be relatively “isolated” in the neuropil to allow its best visualization; (5) dendrites should have defined borders and, as much as possible, be tapering after branching or at distal locations; and (6) dendritic spines should be visible and morphologically distinct than unspecific silver precipitated in the section background.

The general morphology of PC neurons was studied at 260× (using an objective planapochromatic lens UPlanSApo 0.6 NA, Olympus, Japan) using a light microscope (Olympus BX-61, Japan) equipped with a *z*-stepping motor and coupled to a CCDDP72 high-performance camera (Olympus, Japan). Each image was acquired after advancing 0.5 μm for each *z* stack, under high resolution (1360 × 1024 pixels), and submitted to dynamic deconvolution using the Image Pro Plus 7.0 software (Media Cybernetics, United States) during the acquisition process (Dall’Oglio et al., 2013; Reberger et al., 2018). Files were recorded as .TIFF files. The selected images were converted to 8-bit monochromatic pictures before processing (Correa-Júnior et al., 2020). We initially elaborated a 2D reconstruction of the cell body and dendrites of Golgi-impregnated neurons by summing microscopic images at sequential focal planes. Small adjustments of brightness and background contrast were made in final reconstructed images using Adobe Photoshop CS3 software (Adobe Systems, Inc., United States) and/or Neuromantic free software (v1.6.3 programmed in Borland C++ Builder, University of Reading, United Kingdom), without altering the original neuronal features.

Based on this 2D general shape reconstruction, we proceeded next to the 3D reconstruction of selected neurons in accordance with the procedure described in Correa-Júnior et al. (2020). That is, we used the Neuromantic software (as mentioned above), and a semi-automatic tracing of the cell body and dendrites was done for the original stack of microscopic images acquired along with the three spatial coordinates. Reconstructions were achieved as a sequence of 3D points with an ASCII-based format representing dendritic trees as a series of connected cylinders of varying radii identified by orthogonally lines from edge-to-edge (Myatt et al., 2012). The luminosity was inverted to evidence the dendritic shafts details contrasting with the background. Contrast was adjusted for the visualization of thin branches using the algorithm and image processing described in Myatt et al. (2012). Final reconstructions were saved as SWC format (Parekh and Ascoli, 2013). Morphometric data were obtained using the L-Measure free software (Scorcioni et al., 2008) on the 3D reconstructed images. Values were calculated for the cell body length, main diameter and volume, the dendritic diameter of the primary shafts, total number of branches (i.e., the sum obtained starting from primary dendrites, including segments between branching points, and toward the end of main or collateral branches), total length, and total volume of the dendritic tree. However, it must be mentioned that measurements of the neuronal cell body and dendrites can be affected by the fixation procedure and the tissue shrinkage due to each technique used. The morphometric values shown in Figure 8 might not be the actual ones (as occurs *in vivo*) due to unavoidable changes in the nervous tissue following death and the various steps for the present histological processing (Dall’Oglio et al., 2010, 2013, 2015; Reberger et al., 2018; see also Zeba et al., 2008 for additional discussion).

The 3D reconstruction of dendritic spines was done using brightfield images acquired at a final magnification of 1300× using an 100× oil immersion objective lens (planapochromatic UPlanSApo 1.4 NA, Olympus, Japan). Each image was saved with high resolution (2070 × 1548 pixels) and submitted to dynamic deconvolution using the Image Pro Plus 7.0 software. Spines were imaged from proximal to distal branches. Data were obtained by controlling the focus in the *z* axis and acquiring *z*-stacks at sequential 0.1 μm steps. Corresponding images were stored as .TIFF files and converted to 8-bit monochromatic pictures. Each spiny dendritic segment imaged consisted of approximately 100–200 sequential frames (Correa-Júnior et al., 2020).

Following Reberger et al. (2018), spines were 3D reconstructed using an algorithm processed in the MATLAB software (R2105b, The MathWorks, United States). I.e., “after processing the gray scale slices independently or using a median 3D filters in smaller subvolumes, images were processed using the following steps: (a) outlier removal; (b) edge enhancement using a variant of the ‘unsharp masking method’ and image filtering approach based on domain transforms (‘edge-aware’); (c) binarization using an adaptive thresholding approach; (d) pruning false positives (i.e., correction for the maintenance or removal of small objects in the image of interest if they are a dendritic segment and spine or an obvious artifact); (e) 2D flood-fill operation for each slice of the binary volume; (f) tricubic interpolation to smooth transitions between adjacent slices; and (g) visualization of the final volume of the sampled images containing the selected dendritic shafts and their spines” (Vásquez et al., 2018) using the “Fiji” Image J software (Schindelin et al., 2012) with the ‘‘Volume Viewer’’ plug-in.^1^ Images had final adjustments of brightness and contrast made in Photoshop CS3 without altering spine counting or classification (Correa-Júnior et al., 2020).

The identification and classification of each type of 3D-reconstructed dendritic spine was based on previous descriptions (Fiala and Harris, 1999; Arellano et al., 2007a,b; Brusco et al., 2014; González-Ramírez et al., 2014; Dall’Oglio et al., 2015; Vásquez et al., 2018; Correa-Júnior et al., 2020; Rasia-Filho et al., 2021). By rotating the reconstructed images, spines were observed at different angles to determine occurrence from proximal to distal dendrites, number, shape, and size (Reberger et al., 2018). For each spine, we considered: (1) the presence, length, and diameter of a neck, (2) the number of protrusions from a single stalk, (3) the head diameter, and (4) the head shape. According to these morphological features, spines were classified as (1) thin (2) stubby, (3) wide, (4) mushroom-like, (5) ramified, and (6) with a transitional aspect between these classes or as “atypical” (or “multiform”) spines with more complex and varied shapes (Dall’Oglio et al., 2015 and references therein). Tiny protrusions extending from the head of a spine were classified as spinules (Brusco et al., 2014; Zaccard et al., 2020; Petralia et al., 2021).

All computational procedures were run using Windows Microsoft^®^ (version 10), Intel^®^ Core*™* i7-8750H CPU @2.20 GHz, 16.0 GB RAM memory, NVIDIA^®^ GeForce GTX 1050 Ti with 4 GB for image processing.

## Results

The Nissl-stained sections served to identify the cortical cytoarchitectonics and the cell body shape of neurons in layer V in the anterior and central regions of the human PC (Figures 2, 3). Our interest was initially centered in Nissl-stained cells with an elongated spindle-shaped or rod-shaped cell body with two symmetric, vertically oriented primary dendritic shafts emerging at opposite somatic poles. The cell body shape of these cells should be similar to those reported previously for putative VENs (e.g., Nimchinsky et al., 1995; Allman et al., 2005, 2010; Hodge et al., 2020). We observed these cells in the PC layer V and in the transition to layer VI close to pyramidal neurons, which ranged from small and intermediate to large size, and glial cells (Figures 2C, 3C). Nissl-stained spindle-shaped neurons displayed a longitudinal cell body length similar to some neighboring pyramidal neurons (Figures 2D, 3D) as well as the characteristic neuronal chromatin aspect and a prominent nucleolus (Figure 3E).

**FIGURE 4:**
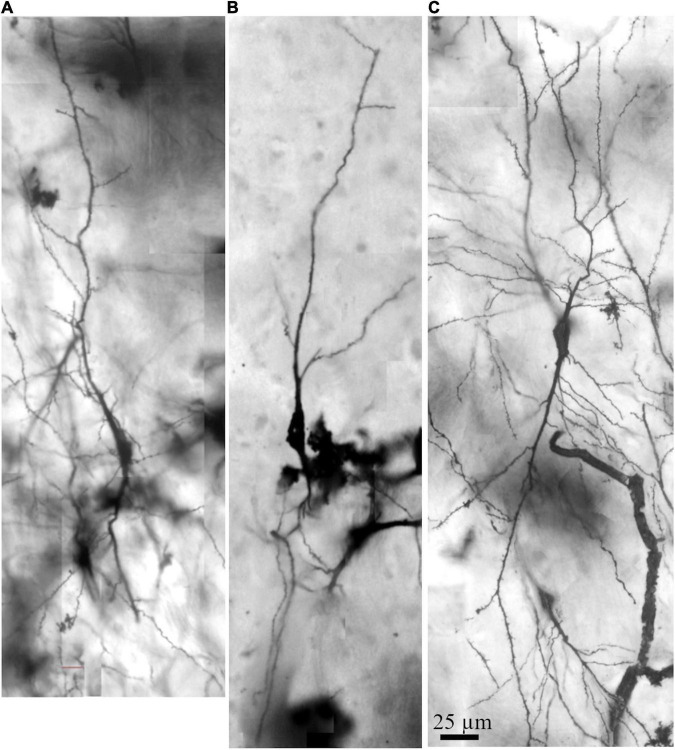
Photomicrographic composition of Golgi-impregnated spindle-shaped neurons from layer V and in the transition to layer VI in the human precuneus observed using brightfield microscopy. **(A–C)** Neurons show a spindle-shaped soma with vertically oriented main primary dendritic shafts at opposite poles and the dendritic ramification in one focal plane. These neurons were 2D and 3D reconstructed and are shown in Figures 5–8 and Supplementary Figures 1–3. Dendritic spines are not quite visible at this magnification. Image adjustment of contrast and brightness made with Photoshop CS3 (Adobe Systems, Inc., United States).

**FIGURE 5:**
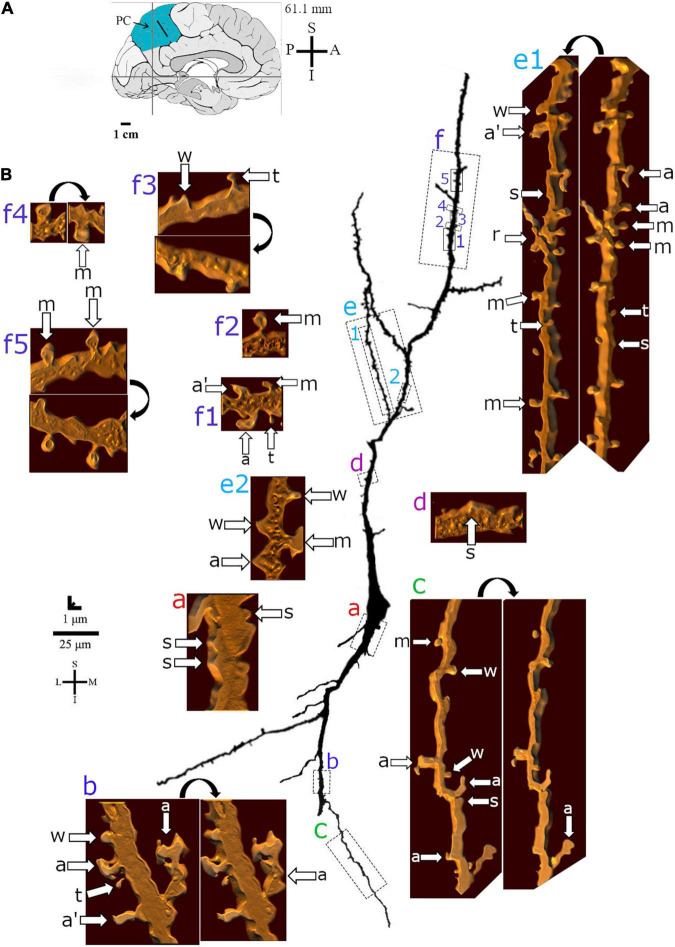
**(A)** Left: Schematic drawing of the medial view of the human brain showing the location of the precuneus (PC, highlighted in blue), central region, 61.1 mm posterior to the midpoint of the anterior commissure. Adapted from Mai et al. (2008, 2016). **(B)** Two-dimensional (for the overall shape) and 3D (for the dendritic and spine details) image reconstructions of serial brightfield photomicrographs of a Golgi-impregnated spindle-shaped neuron from layer V in the human PC (pial surface at the top). Note the cell body shape, the main ascending and descending primary dendrites with a straight course and few ramifications. Proximal to distal dendritic segments (identified by colored letters from “a” to “f”) were sampled and their spines are shown at higher magnification in the adjacent corresponding boxes. Note the distribution of low to moderate density of spines as well as the variety of their shapes. Spines were classified as stubby (s), wide (w), thin (t), mushroom (m), ramified (r), with a transitional (t), or atypical aspect (a). Spine types are indicated by arrows at different rotating angles. An apostrophe with the corresponding spine indicates the presence of a spinule. Image adjustment of contrast made with Photoshop CS3 (Adobe Systems, Inc., United States). Coordinates in **(A,B)**: I, inferior; L, lateral; M, medial; S, superior. Scale = 25 μm for the 2D reconstruction and 1 μm for the 3D reconstructions.

**FIGURE 6:**
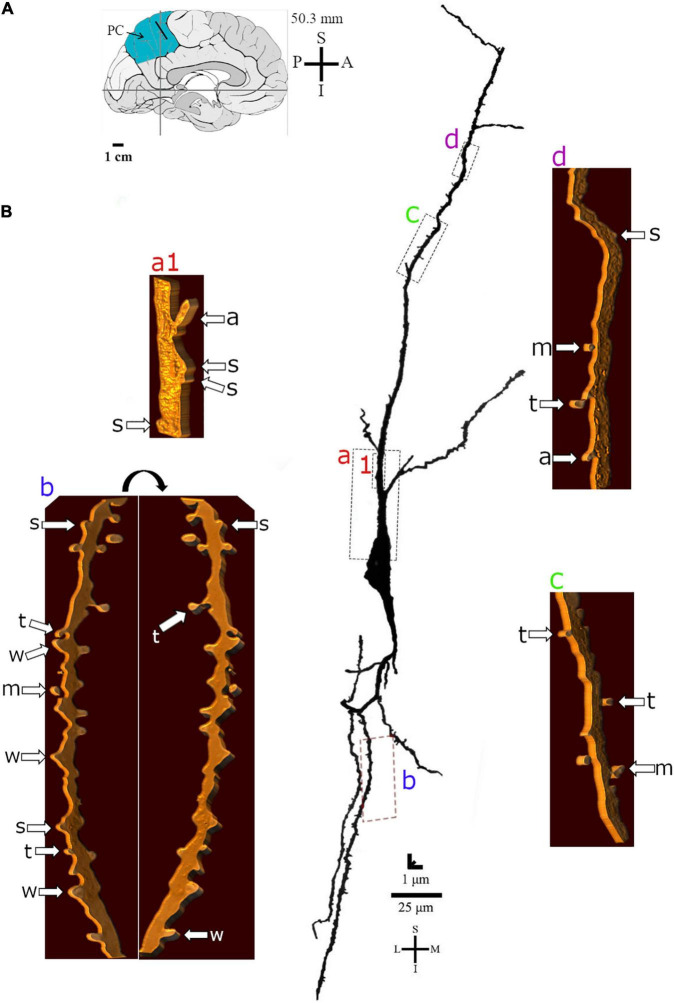
**(A)** Left: Schematic drawing of the medial view of the human brain showing the location of the precuneus (PC, highlighted in blue), anterior region, 50.3 mm posterior to the midpoint of the anterior commissure. Adapted from Mai et al. (2008, 2016). **(B)** Two-dimensional (for the overall shape) and 3D (for the dendritic and spine details) image reconstructions of serial brightfield photomicrographs of a Golgi-impregnated spindle-shaped neuron from layer V in the human PC (pial surface at the top). Note the cell body shape, the main ascending and descending primary dendrites with a straight course and ramifications. Proximal to distal dendritic segments (identified by colored letters from “a” to “d”) were sampled and their spines are shown at higher magnification in the adjacent corresponding boxes. Note the sparse distribution and density of spines. Spines were classified as stubby (s), wide (w), thin (t), mushroom (m), with a transitional (t), or atypical aspect (a). Spine types are indicated at different rotating angles. Image adjustment of contrast made with Photoshop CS3 (Adobe Systems, Inc., United States). Coordinates in **(A,B)**: I, inferior; L, lateral; M, medial; S, superior. Scale = 25 μm for the 2D reconstruction and 1 μm for the 3D reconstructions.

**FIGURE 7:**
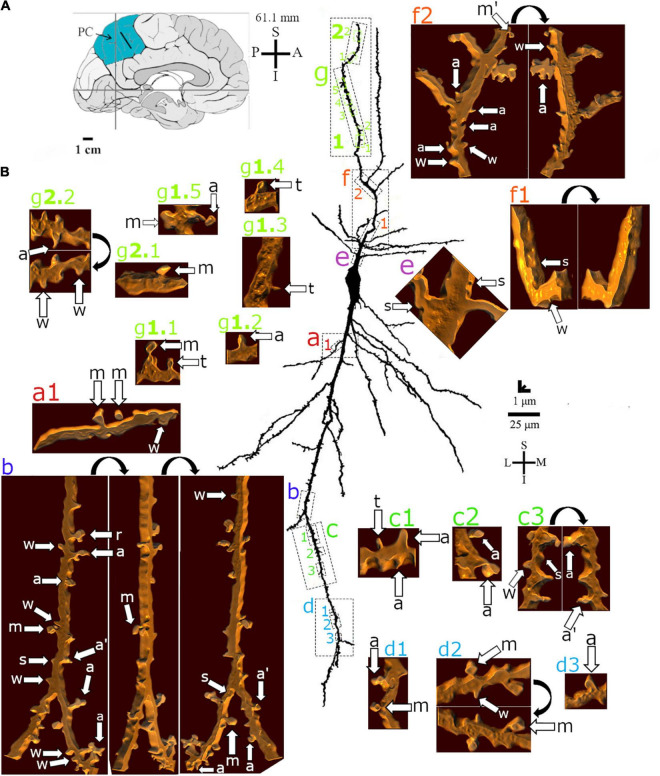
**(A)** Left: Schematic drawing of the medial view of the human brain showing the location of the precuneus (PC, highlighted in blue), central region, 61.1 mm posterior to the midpoint of the anterior commissure. Adapted from Mai et al. (2008, 2016). **(B)** Two-dimensional (for the overall shape) and 3D (for the dendritic and spine details) image reconstructions of serial brightfield photomicrographs of a Golgi-impregnated spindle-shaped neuron in the layer V in transition to layer VI in the human PC (pial surface at the top). Note the cell body shape, the main ascending and descending primary dendrites with more profuse branching points and radial ramifications. Proximal to distal dendritic segments (identified by colored letters from “a” to “g”) were sampled and their spines are shown at higher magnification in the adjacent corresponding boxes. Note the distribution of low to moderate density of spines as well as the variety of their shapes. Spines were classified as stubby (s), wide (w), thin (t), mushroom (m), ramified (r), with a transitional (t), or atypical aspect (a). Dendritic segments and spine types are indicated at different rotating angles. An apostrophe with the corresponding spine indicates the presence of a spinule. Image adjustment of contrast made with Photoshop CS3 (Adobe Systems, Inc., United States). Coordinates in **(A,B)**: I, inferior; L, lateral; M, medial; S, superior. Scale = 25 μm for the 2D reconstruction and 1 μm for the 3D reconstructions.

**FIGURE 8:**
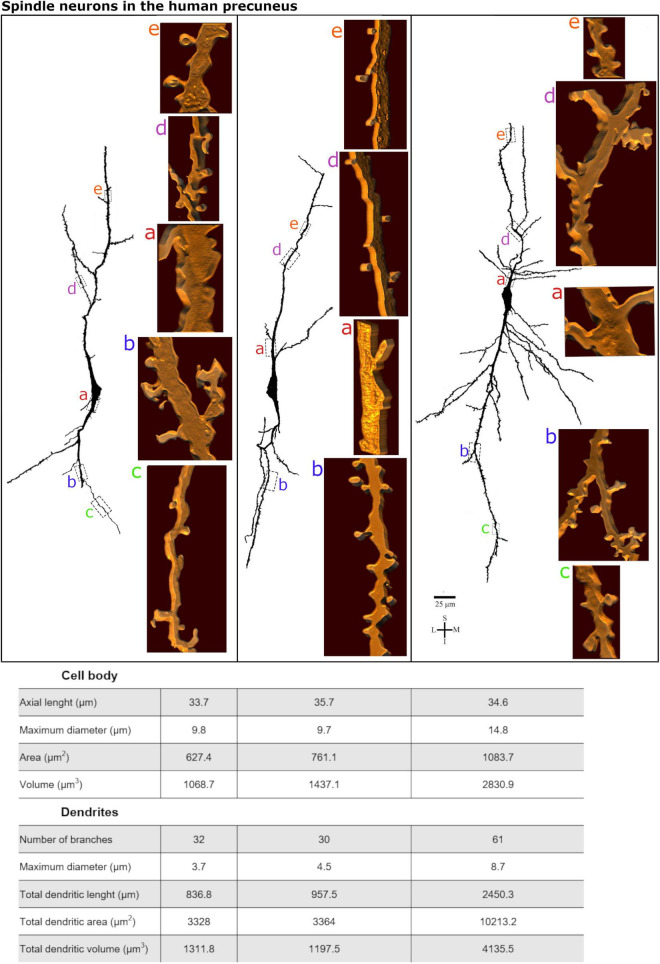
**(Top)** Comparison of the morphological features for the dendrites of spindle-shaped neurons in the human precuneus cortex shown in Figures 5–7. Compare the branching pattern of the spindle-shaped cell in the right with the others. Pleomorphic spines are shown from proximal to distal segments in these cells. Image adjustment of contrast made with Photoshop CS3 (Adobe Systems, Inc., United States). **(Bottom)** Quantitative data were obtained from these cells. Morphometrical data refer to the cell body parameters (note the similar axial length) and features of both ascending and descending dendrites (note the values for the neuron in the right compared to the others).

**FIGURE 9:**
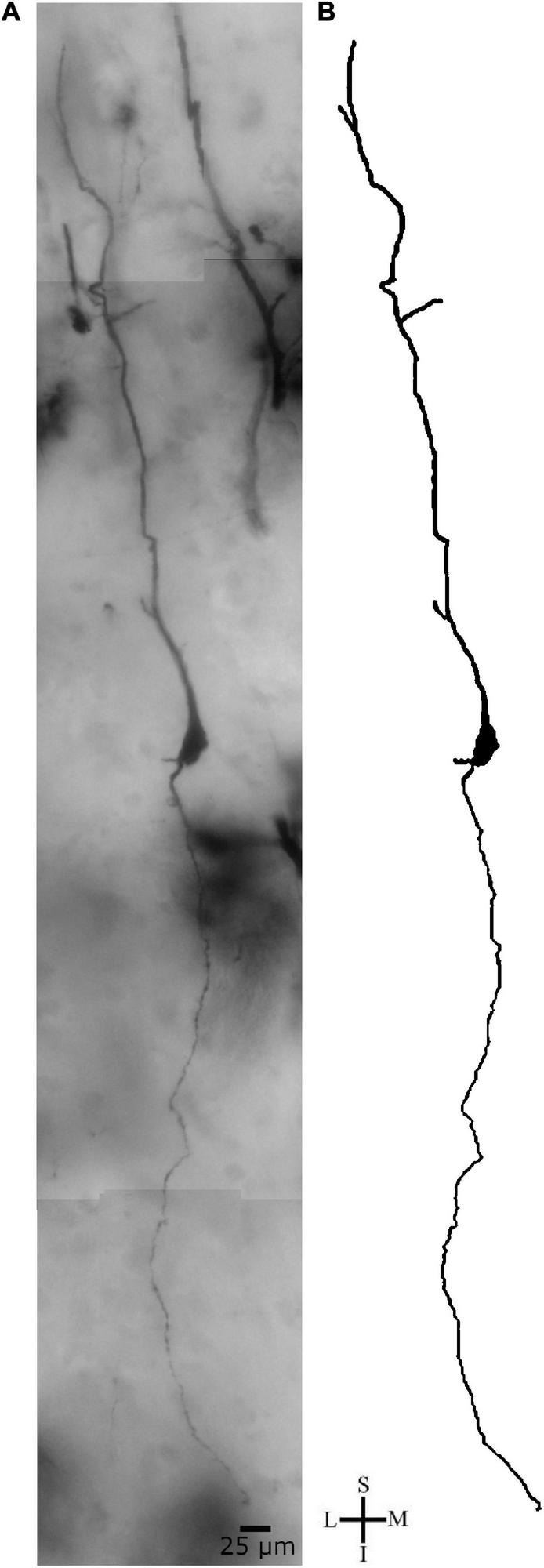
**(A)** Photomicrographic composition of a Golgi-impregnated spindle-shaped neuron from layer V in the transition to layer VI in the human precuneus (central region, 61.1 mm posterior to the midpoint of the anterior commissure) observed using brightfield microscopy. **(B)** Two-dimensional image reconstruction of the same neuron in **(A)** (pial surface at the top). Note the cell body shape and the two main ascending and descending primary dendrites with rare branching points. Image reconstructed using the Neuromantic software (University of Reading, United Kingdom) with adjustment of contrast made with Photoshop CS3 (Adobe Systems, Inc., United States). Coordinates in **(A,B)**: I, inferior; L, lateral; M, medial; S, superior. Scale = 25 μm.

**FIGURE 10:**
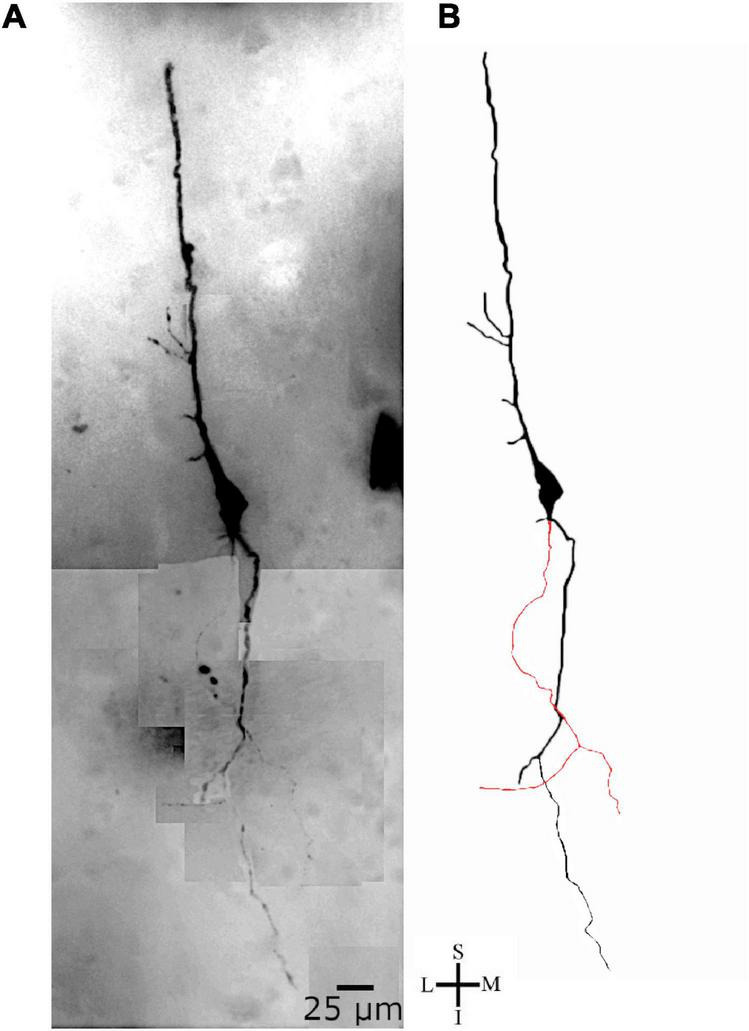
**(A)** Photomicrographic composition of a Golgi-impregnated spindle-shaped neuron from layer V in the transition to layer VI in the human precuneus (central region, 61.1 mm posterior to the midpoint of the anterior commissure) observed using brightfield microscopy. **(B)** Two-dimensional image reconstruction of the same neuron in **(A)** (pial surface at the top). Note the cell body shape, two main ascending and descending primary dendrites with various branching points, and the presence of a delicate axon (colored in red) emerging from the soma, close to the descending dendrite, and ramifying at approximate right angles. Image reconstructed using the Neuromantic software (University of Reading, United Kingdom) with adjustment of contrast made with Photoshop CS3 (Adobe Systems, Inc., United States). Coordinates in **(A,B)**: I, inferior; L, lateral; M, medial; S, superior. Scale = 25 μm.

**FIGURE 11:**
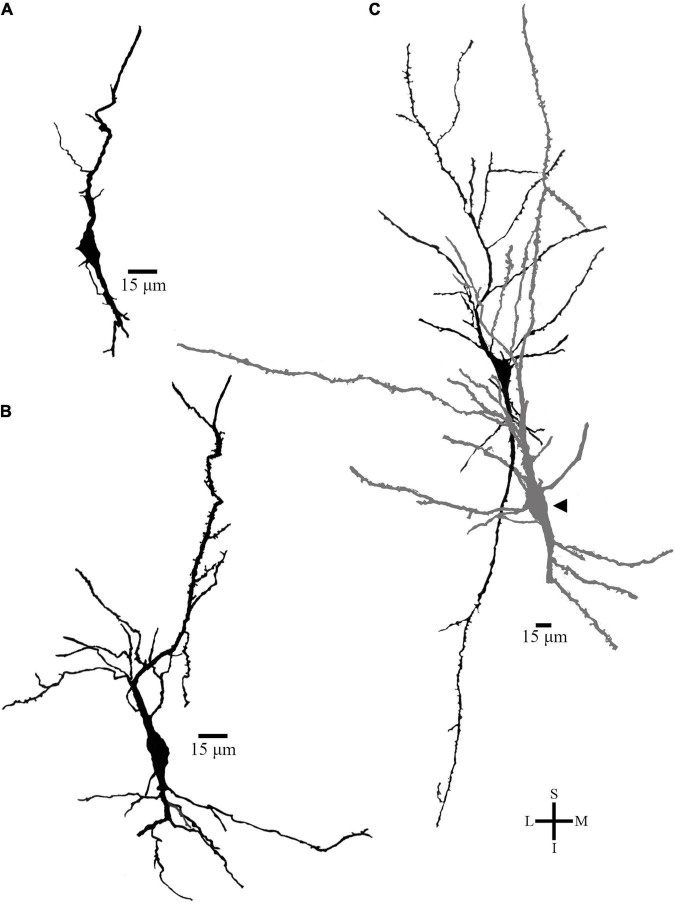
**(A,B)** Two-dimensional image reconstruction of serial brightfield photomicrographs showing the overall shape of Golgi-impregnated neurons from the pleomorphic layer VI in the human precuneus (central region, 61.1 mm posterior to the midpoint of the anterior commissure, pial surface at the top). Note the fusiform cell body shape, short ascending, and descending primary dendrites with variable ramification and collateral branches. In **(C)**, a different fusiform cell (in dark) displays two main primary dendritic shafts but also some additional small primary branches that alter the shape of the cell body. The arrow points to a “modified pyramidal neuron” (in gray) according to Braak (1980). Note the scale bar for this figure and compare it to the ones presented for layer V spindle-shaped neurons in this same brain area (Figures 5–8). Image adjustments made with Photoshop CS3 (Adobe Systems, Inc., United States).

**FIGURE 12:**
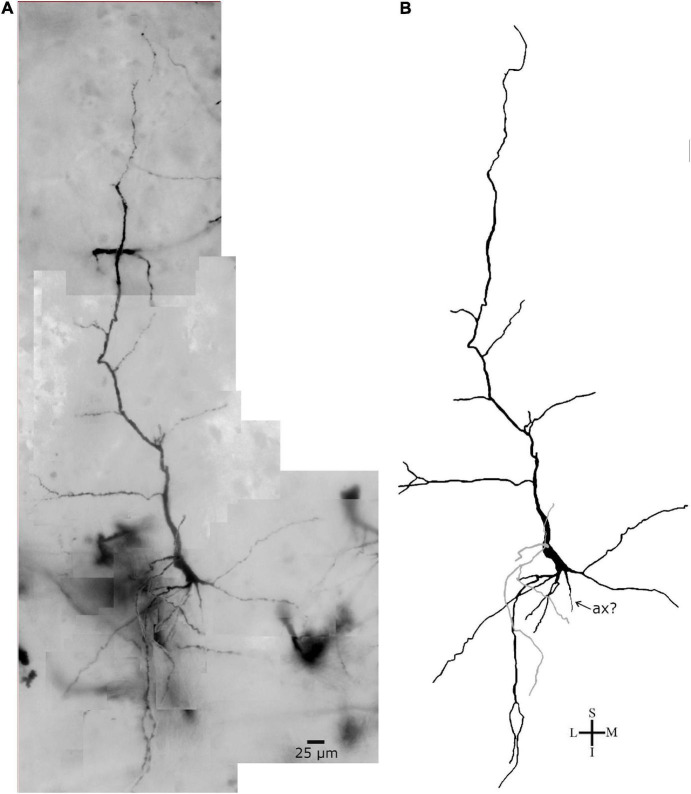
**(A)** Photomicrographic composition of a Golgi-impregnated spindle- to rod-shaped neuron from the inner layer VI in the human precuneus (central region, 61.1 mm posterior to the midpoint of the anterior commissure) observed using brightfield microscopy. **(B)** Two-dimensional image reconstruction of the same neuron in **(A)** (pial surface at the top). Note the elongated cell body shape, two main longitudinally oriented primary dendrites with various branching points, a third thinner primary dendrite arising from the cell body (colored in gray). Note the thick aspect of the descending primary dendrite, its ramification in secondary branches, and a process that would be an axon hillock (ax, indicated by an arrow). The axon extent or its ramification are not visible. Image reconstructed using the Neuromantic software (University of Reading, United Kingdom) with adjustment of contrast made with Photoshop CS3 (Adobe Systems, Inc., United States). Coordinates in **(A,B)**: I, inferior; L, lateral; M, medial; S, superior. Scale = 25 μm.

There are limitations for the Golgi method when studying adult human *postmortem* samples. That is, not all cells were completely impregnated in the human PC, and it was not possible to reliably determine the presence of an axon and its ramification in all neurons. Few well-impregnated spindle-shaped neurons randomly fulfilled the inclusion criteria for further study. Descriptive data are provided for these available neurons without further statistical comparisons. The three subjects studied here had neurons in layer V showing the spindle-shaped soma and two primary dendrites, but few cells were well-impregnated. We selected the Nissl staining and the best Golgi-impregnated spindle-shaped neurons that we could obtain, shown in Figures 2–10 and Supplementary Figures 1–3 from case #4 (described in Table 1). For example, in 7 serial sections from this specific case, we found 15 randomly impregnated pyramidal neurons (one example is shown in Supplementary Figure 4, most of the others were not completely impregnated) and 8 spindle-shaped cells (5 of them are shown here, the others had only the cell body and short “cut-off” proximal dendritic branches visible). The sampled spindle-shaped neurons were observed along the anterior or the central parts of the PC (see legends of the corresponding Figures 2–10). We also observed some small fusiform and other pleomorphic neurons, including a spindle- to rod-shaped cell, in layer VI of the human central part of the PC (Figures 11, 12), as described below.

Using the Golgi results, we performed the 3D reconstruction of three spindle-shaped neurons found in layer V and in the transition to layer VI to evidence their dendritic and spines features in the human PC (Figures 4–8). The characteristic aspect of the cell body at different viewing angles and the preferred vertical orientation of the two main dendritic shafts of these cells are shown in Supplementary Figures 1–3. These neurons have a similar cell body shape, but show some differences in the dendritic branching pattern (Figures 4–8). That is, although they have two main longitudinal ascending and descending shafts with a straight course, dendrites varied in the number and aspect of the collateral branches (Figure 8 for comparison). Spindle-shaped neurons can have few collateral dendritic branches with an oblique orientation (Figures 5, 6) or display more profuse ramification in both ascending and descending dendrites and collateral branches with a higher radial extension (Figure 7). Morphometric data were obtained to exemplify the present observations (Figure 8). It is also worth noting that the spindle-shaped neurons shown in Figures 5–7 required 54, 142, and 31 serial *z* stacks (0.5 μm each) for their 3D reconstruction, respectively (Supplementary Figures 1–3). That is, these neurons can display a 3D dendritic extension restricted to the same vertical axis where the cell body is or can also exhibit obliquely oriented dendritic branches with a higher extension toward the surrounding volume. For example, the spindle-shaped neuron shown in Figure 6 has more branches along the *z*-axis (and therefore needed more *z* stacks for its imaging), which is observed at different rotation angles after 3D reconstruction (Supplementary Figure 2).

Dendritic spines showed a variety of shapes and sizes intermingled in the same dendritic segments (Figures 5e1, 6b,d, 7b). Spine types ranged from small to large (Figures 6a1,b) with stubby, wide, thin, mushroom, ramified, transitional aspects or more complex shapes with different neck thickness and/or multiple bulbous structures (Figures 5e1, 7f2 right).

Mushroom spines showed heads with a bulbous or a perforated-like aspect (Figure 5e1, left). Spines with transitional or atypical aspects showed diverse shapes (Figures 5b,c, 7b,c2,d1). Among them, there is a double spine with a neck and a bulb followed by a second neck giving rise to another ending bulb (Figure 5c, left image, “a” in the right side of the dendrite). Pleomorphic spines were found from proximal to distal ascending and descending dendrites, showing a sparse to moderate density toward more distal segments (Figures 5–7). Spines occurred either isolated or grouped (Figures 5b,c, 7b,f2) in the main and collateral dendritic branches (Figures 5b,e, 6b,c, 7b–d,g). Spinules were also observed in different spine types (Figures 5e1,f1, 7b,c3).

There were some additional features for the spindle-shaped neurons in layer V and in the transition to layer VI in the human PC. We have also observed a spindle-shaped neuron (longitudinal length and higher diameter of 37 and 19 μm, respectively) with straight dendritic branches devoid of main ramifications in both ascending and descending branches and with a moderate density of small spines (Figure 9), and another spindle-shaped neuron (longitudinal length and higher diameter of 38 and 19 μm, respectively) with a delicate axon emerging from the cell body, close to the descending primary dendrite, and with few spines along the dendritic shafts (Figure 10). The axon in this latter cell emerges from a short hillock, is directed to the inner cortical layer, has a tiny aspect and few ramifications at approximate right angles (Figure 10).

Lastly, Golgi-impregnated cells with varied cell body shapes occur in the polymorphic layer VI of the human PC (Figures 11A,B). Some fusiform cells are smaller than layer V spindle-shaped neurons (compare the scale bar in Figure 11 and in Figures 5–7). For example, the cell body of two small fusiform neurons, measured after the cellular 2D reconstruction, had 18 and 20 μm for the longitudinal length and 8 and 9 μm for the higher diameter (Figures 11A,B, respectively). These neurons also show two vertically oriented primary dendrites arising from opposite somatic poles, but very short dendritic branches with a restrict extension in the neuropil (Figure 11A). Some of the thin collateral branches radiate in various angles and show an oblique to horizontal projection (Figure 11B). Other fusiform cell displays two main primary dendritic shafts but also other small primary branches that alter the shape of the cell body (Figure 11C, neuron in dark). This latter cell was observed adjacent to a “modified pyramidal neuron” (MPN, according to Braak, 1980; Figure 11C, arrow head) in this inner layer. Interestingly, an elongated spindle- to rod-shaped neuron (longitudinal length and higher diameter of 41 and 16 μm, respectively) was found at the deeper part of layer VI in the PC (Figure 12). This spiny cell has two thick primary dendrites. The descending one is thicker than the ascending shaft, gives rise to two secondary branches with radially oriented collaterals, and a segment that appears to be the axon hillock. The ascending dendrite has a longer extension and some collaterals that radiate to the adjacent neuropil. There is a third dendritic branch, thinner than the others, emerging from the cell body and directed to the top of the section (Figure 12).

## Discussion

We describe the presence and the morphological features of Nissl-stained and Golgi-impregnated spindle-shaped neurons in the anterior and central regions of the PC in the human PMC. They were found close to pyramidal cells in the cortical layer V and in the transition to pleomorphic layer VI. In the PC, these spindle-shaped neurons display a similar cell body shape and two primary dendritic branches with a longitudinal spatial orientation as putative VENs described in other human brain areas (Nimchinsky et al., 1995; Allman et al., 2005, 2010; Watson et al., 2006; Fajardo et al., 2008; Cobos and Seeley, 2015; Raghanti et al., 2015). Nevertheless, the neurochemical and transcriptomic identity of these cells have to be determined to establish their definite classification. Here, we will discuss the morphology of VENs and critically compare our Golgi data with those from other authors (Watson et al., 2006; Banovac et al., 2019, 2021) as well as with other congruent approaches that have been used to identify human VENs in the ACC and FI. These PC layer V spindle-shaped neurons showed few dendritic branches in the main ascending and descending dendritic shafts or a more ramified aspect with collateral dendrites at different angles and extension along the surrounding neuropil. In addition, dendritic spines ranged from sparse to moderate from proximal to distal segments. We observed intermingled spines of varied shapes and sizes, as well as the presence of spinules after 3D reconstruction. These results would add to the cytoarchitecture and to the synaptic and information processing in the human PC integrated in multimodal networks relevant for the DMN and general intelligence (*g*) in the human brain. These findings also underscore the need for an in-depth characterization of these spindle-shaped (or putative VENs) in both healthy individuals and in neurological and psychiatric conditions involving the PC in the context of the PMC functioning, as commented below.

### Neurons Might Be Classified as Von Economo Neurons by Concurring Techniques

There is an important current discussion on what can be considered a VEN based on morphological criteria (Nissl and Golgi techniques), after using *in situ* hybridization and immunohistochemistry assays for VEN-associated distinctive expression of cellular markers and/or by employing single nucleus RNA sequencing to obtain transcriptome data and to predict cellular functional properties. In a recent paper, Banovac et al. (2019) defined “VENs on Golgi staining as a neuron with the following morphological features: an elongated, stick-like cell body gradually continuing into thick apical and basal stem, a brush-like basal stem arborization and an axon origin distant from the cell body,” i.e., at least 100 μm away from the cell nucleus. These authors also recommended that “the identification of von Economo’s specialized cells in other cortical regions and non-primates should be done by demonstrating the dendritic and axonal morphology or by identifying specific markers or marker combinations that would enable the identification of VENs without relying solely on morphology” (Banovac et al., 2021).

Let us comment on the current data that include the cell body, the dendritic and axonal morphology with the identification of VEN markers focusing in cells from the layer V of the human ACC and FI. We will exclude small fusiform or MPNs in layer VI from the present discussion. First, it is important to consider that von Economo and Koskinas identified “VENs” in humans using the Nissl staining (see Figures 4 and 5 in Banovac et al., 2021). That is, it was the cell body shape, its relative size compared to adjacent cells, and the aspect of two main primary dendrites of these neurons located in the layer V of restricted brain areas that led to the identification of “VENs as VENs.” The “stick- or corkscrew-shaped cell body were clearly distinguishable from other spindle-shaped cells found throughout the cerebral cortex” (Banovac et al., 2021). This cell body shape described by von Economo and Koskinas resembles the elongated soma of neurons previously drawn by Ramón y Cajal (Figure 1 from Banovac et al., 2021). However, it is also possible to observe a certain variability in the cell body shape of VENs drawn by Ramón y Cajal, including a likely “spindle” aspect (compare the cell body shape and the descending dendrites originating an evident axon in the four VENs marked as C and D in Figure 1 from Banovac et al., 2021; data from the FI of a 1-month-old human girl). Afterward, Nimchinsky et al. (1995) described Nissl-stained spindle neurons in the layer Vb of the human ACC with “…a basal dendrite that was at least as thick as its apical dendrite….” These “spindle neurons were readily distinguishable from pyramidal neurons and exhibited a variety of morphologies. Some were very slender and elongate, with apical and basal dendrites nearly as thick as the soma at its widest point. Others were shorter, more stout, and usually curved. Occasionally, neurons were encountered with a bifid basal dendrite or a third major dendrite emerging from the soma. In addition, lipofuscin deposits were common and were occasionally so large that they distorted the shape of an otherwise very slender neuron… The significance of this cellular variability is not clear, but it might be related to the cytoarchitectonic variability in this region…” (see additional examples in Figure 4D from Jacot-Descombes et al., 2020).

While maintaining the particular elongated somatic aspect and two main primary dendrites at opposite somatic poles, some variability for the rod and spindle shapes of the cell body of putative VENs in cortical layer V could be observed using additional techniques in human samples (e.g., for transcriptomic-related data, see Figure 1a, right image, and Figures 2b and 2c in Hodge et al., 2020; Figure 1A for the rod and spindle cell body shape of neurons expressing specific genes shown in Figures 4B′ and C′ in Yang et al., 2019; for the different immunocytochemical stainings of VENs, including cell body shape and primary dendrites, see Figure 3 in Allman et al., 2005; the cell body variability in Figures 10B and 11B in Allman et al., 2010; the somatic shape and primary dendrites in Figures 3D and 3F as well as the immunohistochemistry images in Figures 4D and 4F in Dijkstra et al., 2018; and, the immunostaining pattern shown in Figures 2A and 2B in Stimpson et al., 2011). Human PC neurons are also examples of some spindle-shaped cells in the human brain in Figure 3A (Correa-Júnior et al., 2020), and this cell body feature directed further study of the ACC VENs shown in Figures 3B–D and Figures 4–8 in this report. Also worth noting are the cell body shape of a NeuN/SMI-32 double-immunolabeled VEN shown in Figure 11B in Banovac et al. (2019), the cell body shapes of presumed VENs after *in situ* mRNA hybridization (and high level expression) of FEZF2 in the FI layer V (Figure 4D″ in Cobos and Seeley, 2015), and the fact that, using Golgi-Cox sections to identify VENs in the deep part of layer V and the upper part of layer VI in the human ACC, “most VENs had a more spindle-shaped cell body, and the point of demarcation between the soma and the stems was more apparent” (Banovac et al., 2019).

These findings would accompany the distinction made when classifying VENs, bipolar ovoid and rhomboid-shaped cell bodies or, if one considers the various results for putative VENs in cortical layer V, the identification of human VENs with a cell body shape with some variability between stick-like or rod and spindle shapes, for example. There is also some similarity between the cell body shape (used as the morphological parameter in this case) of a layer V “VEN” included in the original study of von Economo and Koskinas (shown in the bottom left of Figure 4 in Banovac et al., 2021) and the “bipolar” neuron (not considered a “VEN”) shown in Figure 10A (leftmost neuron) from Banovac et al. (2019). A parallel discussion could be done on VENs shapes and markers in the macaque monkey (e.g., see Figures 2A and D–G, and Figures S1 E′, F′, and F″ in Evrard et al., 2012). Moreover, the 3D reconstruction is a useful procedure that can provide complementary data to 2D images. When observed at different rotation angles, the cell body shape of the same 3D reconstructed Golgi-impregnated VEN can show a spindle-shape or a slender aspect (for example, see the three Supplementary Figures in 3D animation in Correa-Júnior et al., 2020 and the present Supplementary Figures 1–3).

Von Economo neurons, fork cells, and a subset of pyramidal cells are transcriptomically similar to one another in the human FI (Hodge et al., 2020), and layer V VENs express some markers that can also be found in fork cells and large pyramidal neurons in the human ACC or FI (Allman et al., 2010; Stimpson et al., 2011; Dijkstra et al., 2018). A within cell class phenotypic variability may occur for VENs since not all of them are immunolabeled by the same marker at the same time in the FI layer V (see Figure 11B in Allman et al., 2010; Cobos and Seeley, 2015). These data would question how layer V VENs are a morphological diversification of an evolutionarily conserved cell type, a morphological modification of pyramidal neurons (as a spindle-transformed cell) or represent a regionally distinctive and selectivelly evolved specialized cell type in the human cerebral cortex (Allman et al., 2005; Cobos and Seeley, 2015; Banovac et al., 2019, 2021; Yang et al., 2019; Hodge et al., 2020).

Notably, thick-tufted layer V pyramidal neurons are a heterogeneous class of cells, but are morphologically different from their neighboring VENs (Watson et al., 2006; compare with Ramaswamy and Markram, 2015; Cembrowski and Spruston, 2019; Rasia-Filho et al., 2021); see also comments on the likely coexistence of discrete and continuous variations that underlie cell-type diversity in BRAIN Initiative Cell Census Network (BICCN), 2021. VENs emerge mainly after birth, increase in number until age 4 years (Allman et al., 2005), and can be susceptible to alterations in some neuropsychiatric disorders (as described below). Compared to pyramidal neurons, a recent transcriptomic profile study identified 344 genes with VEN-associated expression in the human ACC, including 215 higher and 129 lower expression genes, some for morphogenesis, dendritic branching and axon myelination or for neurological and psychiatric disorders in humans (Yang et al., 2019). Human ACC VENs have four lower expression genes (HPCA, HPCAL1, RALGDS, and NUBP2) that directly interact with RHOA, an important regulator of dendritic morphogenesis (Yang et al., 2019). Accordingly, “the single large basal dendrite of VEN might have resulted from a transformation of the genetic programs during evolution for pyramidal neuron development to modify the basal dendrite in order to concentrate its growth in the primary component and suppress the secondary and tertiary branching” (Yang et al., 2019 and references therein). In these cells, the MEF2C high expression is also expected to reduce the density of dendritic spines, whereas many myelination-related genes (such as MBP and PLP1) showed VEN-increased expression (Yang et al., 2019). This latter would make difficult the reliable identification of the VEN axon and to trace its pathway and ramification after myelinization using the Golgi method, specially in samples obtained from adults. Some of these proposed implications for selective gene expression are supported by the morphological data obtained on dendrites and spines of putative VENs, compared to pyramidal neurons, in the human ACC reported by Watson et al. (2006).

As occurs for the cell body shape, VENs would also display heterogeneity in the dendritic architecture. Banovac et al. (2019) proposed the division of VENs in two groups based on peak total dendritic length, i.e., small VENs with 1500–2500 μm, and large VENs with 5000–6000 μm. Correa-Júnior et al. (2020) suggested a *continuum* of branching patterns. Therefore, it would be interesting to consider how dendrites and spines can alter their shape, even within the same class of neuron, due to region-specific specializations and according to the local processing of the synaptic demand from different neural circuits. For example, there are clear variations in the branching pattern of basal dendrites, the length and distal apical ramification with a tuft aspect if pyramidal neurons are located in the superficial or deeper parts of the layer V in the rat frontal cortex (Morishima and Kawaguchi, 2006). Human pyramidal neurons also show a heterogeneity in dendritic morphology with four different main apical branching patterns in the hippocampal CA1 area (Benavides-Piccione et al., 2020). The axon in these cells can emerge either from the soma (66% of the cases) or from the initial portion of a basal dendrite (44% of the cases; Benavides-Piccione et al., 2020). Therefore, it would be plausible that a certain degree of plasticity and heterogeneity would also occur for VENs in specific areas, including the morphological features of their cell body shape (as mentioned above), dendritic branching, and spine features in the human brain. The 3D reconstruction of Golgi-impregnated VENs located in the layer V of the human ACC indicates a *continuum* of dendritic and spine heterogeneity, ranging from less ramified to more branched ones, also including the presence and density of pleomorphic dendritic spines (Correa-Júnior et al., 2020). As mentioned in Cobos and Seeley (2015), “human VENs may be a heterogeneous population comprising projection neurons with diverse targets including the contralateral cortex.” These are possibilities that deserve further development and interpretation with the available morphological data (Watson et al., 2006; Banovac et al., 2019, 2021; Correa-Júnior et al., 2020).

Let us consider again the fact that the cell body shape and the aspect of the primary dendrites served to identify putative VENs in the layer V of ACC and FI using different histological, neurochemical and transcriptomic markers, although there is not a single specific marker for these cells currently. Watson et al. (2006) used the characteristic cell body shape as one criteria for the identification of layer V human VENs, as follows: “The criteria for classifying a neuron as a VEN was an elongated, large soma in layer 5 of the FI or ACC, a prominent basal dendrite, and symmetrical morphology along the horizontal and vertical axes of the cell…. We further constrained the category to include only those neurons that had no additional dendrites or branching for a half-soma’s distance along the length of the proximal dendrites.” Even with this inclusion criteria, it would be that neurons in the layer V of the ACC shown in Watson et al. (2006) or in Correa-Júnior et al. (2020) are not identical to the MPN in the layer V of the prefrontal cortex (shown in Figure 3, right side, from Banovac et al., 2021). The neuron shown in Banovac et al. (2021) is a spindle-shaped cell with two ascending and descending dendrites devoid of any ramification along their long course, whereas the spindle-shaped neuron in Figure 4 from Correa-Júnior et al. (2020) displays a sparse ramification and no visible axon hillock arising from the cell body, for example. On the other hand, one spindle-shaped neuron in the PC (Figure 9) has both dendrites with a straight course and a scarce radial arborization, apparently more restricted than the neuron shown as a VEN in the ACC by Watson et al. (2006; shown in Figure 4B). This PC neuron would resemble the dendritic features of the neuron classified as a prefrontal MPN by Banovac et al. (2021; shown in Figure 3, right side). Let us also remember that VENs have been considered a subclass of MPNs (Banovac et al., 2019).

The description of the tuft aspect of the descending dendritic branches, from where the axon is observed (Banovac et al., 2019, 2021), is an important morphological feature evidenced in the original Golgi study of VENs, and the efforts to look for these specific cells in the human brain are impressive (Banovac et al., 2021). Notwithstanding, additional images for the descending dendrites in putative VENs have been reported. For example, an elongated rod-shaped neuron, classified as VEN and labeled with an antibody to vasopressin 1a receptor in the ACC of an adult male human, displayed a main descending dendrite with no tuft or brush-like branches (visible along approximately 100 μm in Figure 3a in Allman et al., 2005). The same was found for two layer V rod-shaped and spindle-shaped VENs in the FI of a human male after immunocytochemical staining for DISC-1 (showing a straight descending dendrite visible along approximately 100 μm in the left neuron in the Figure 11B from Allman et al., 2010) and for a rod-shaped neuron stained for neuromedin B (with a straight descending dendrite visible along approximately 125 μm in Figure 10C in Allman et al., 2010). Hodge et al. (2020) provided the transcriptomic evidence that VENs are regionally specialized extratelencephalic-projecting excitatory neurons and included a biocytin-filled putative layer V VEN from the FI of an adult human in their report. This is the first electrophysiological single neuron patch clamp recording for human putative VENs, whose data were obtained from *ex vivo* peri-tumor insula tissue brain slices from a single human donor. These authors described some distinctive intrinsic membrane properties for putative VENs relative to neighboring pyramidal neurons. The local putative VEN had “the expected large spindle-shaped morphology with large caliber bipolar dendrites that extended into layer 6 (descending trunk), as well as toward the pial surface into upper layer 3 (ascending trunk). Dendritic branching was very simple, but with notable short and wispy lateral branches concentrated proximal to the soma. The axon could not be readily distinguished from these finer dendrites” (shown in Figure 5C in Hodge et al., 2020). Considering the likelihood of morphological heterogeneity within the same class of neurons (e.g., as occurs in pyramidal ones, Cembrowski and Spruston, 2019; Benavides-Piccione et al., 2020; Rasia-Filho et al., 2021), it would be plausible to consider that the description of some morphological variability for the dendrites of VENs would not be conflicting; rather, they would be complementary to the original descriptions of these cells. The integration of multiple approaches and criteria are need to reach a consensus in this field (Banovac et al., 2021).

There are few data on the axonal morphology of human VENs (but see relevant figures in Banovac et al., 2019, 2021). The adapted Golgi method used here for human *postmortem* brain (Dall’Oglio et al., 2010, 2015; Vásquez et al., 2018; Rasia-Filho et al., 2021) does not always provide the identification of the axon to trace its origin and ramification, which was also mentioned in Correa-Júnior et al. (2020). We described the diffuse axonal pattern (which would imply that the observed fibers would be not myelinated) in the neuropil of the human medial amygdaloid nucleus, and identified the axon hillock emerging from the cell body or in close primary dendrite in some local cells using this same Golgi technique (Dall’Oglio et al., 2013). However, the aspect of the axon is a limitation in the present study. In our samples (Figures 4–7), we were not confident to determine that a segment with varied tapering aspect would be an axon arising from a distal dendrite without having additional morphological evidence for the axonal diameter, angle of ramification, and caliber after branching (as shown by Ramón y Cajal in Figure 1 from Banovac et al., 2021) or having other supporting data from immunolabeled components of the axonal cytoskeleton, anterograde or retrograde tracing, intracellular dye, and/or electrophysiological recordings.

On the other hand, there was a spindle-shaped neuron in the PC with an axon hillock arising from the cell body (Figure 10). This would characterize it as a MPN according to Banovac et al. (2021). It is very important to determine if all VENs in the human FI and ACC (and, then, for all VENs to be considered true “VENs” in other areas of the human cerebral cortex) must have an axon arising from a brush-like basal stem arborization (Banovac et al., 2019, 2021). Most histological, immunocytochemical, *in situ* hybridization, single nucleus RNA sequencing, and electrophysiological data will have to be reconsidered according to these results and checked for these specific basal dendritic and axonal characteristics. Alternatively, there might be a growing body of evidence in the literature that would indicate that layer V stick-like, rod- and/or spindle-shaped neurons are likely phenotypes of VENs that, independently of the determination of the strict aspect for their dendritic branching pattern, have been congruently reported as specialized cells with functional implications and abnormalities in various neuropathological conditions (see Figure 2 in Seeley, 2008; the morphological aspect of the soma and proximal dendrites of VENs in Figure 7 from Raghanti et al., 2015; Figure 2A and data in Jacot-Descombes et al., 2020; and, Table 2 from Banovac et al., 2021).

The morphological description provided by Banovac et al. (2019, 2021) for VENs is an important piece for studying these cells. From other approaches, some variability in the spindle- to rod-shaped cell body or in the aspect of the descending dendrite of the cells identified as VENs were also obtained and/or are expected to occur. In this regard, the cellular components of the layer V of the PC represent an interesting area open to further research in the human brain. Here, spindle-shaped neurons might represent a spectrum that ranges from a morphology of two straight dendrites and rare ramification (Figure 9) or with an axon arising from the cell body (Figure 10) to a more profuse dendritic ramification, with various collateral branches and a radial extension, and no visible axon hillock in the cell body (Figures 6, 7). There are some hypotheses to be considered. These two former cells would resemble the description of MPNs (Banovac et al., 2021), the others would be a subtype of VENs. It would be also possible that VENs would show heterogeneity in their dendritic features, including “simple” forms observed in the ACC (Watson et al., 2006) to small and large VENs in terms of total dendritic length (Banovac et al., 2019). The possibility that spindle-shaped neurons can be VENs is consistent with the visualization of this cell body type by Ramón y Cajal in the FI. The length and aspect of the descending dendrites of putative VENs would allow some heterogeneity, as observed in rod-shaped neurons with a straight descending dendrite immunolabeled with antibodies to vasopressin 1a receptor, DISC-1, and for neuromedin B (Allman et al., 2005, 2010), after patch clamp recording and intracellular dye injection of a putative VEN with a large spindle-shaped soma and with no descending dendritic tuft (Hodge et al., 2020), and in rod-shaped neurons with a brush-like basal stem arborization and an axon origin distant from the cell body (Banovac et al., 2019, 2021). It is possible that not all spindle-shaped cell body cells be defined as VENs (Banovac et al., 2021), but putative VENs in layer V would be spindle-shaped, rod-shaped, stick, or corkscrew cells with regional specializations as observed with concurring techniques and neurochemical, electrophysiological, transcriptomic, and neuropathological characteristics (Allman et al., 2005, 2010; Raghanti et al., 2015; Yang et al., 2019; Hodge et al., 2020; Jacot-Descombes et al., 2020).

To advance this field, *Patch-seq* transcriptomes, a method relying on sequencing somatic RNA of single patch-clamp-recorded neurons, would help to identify if neuron types previously considered homogenous would be set into distinct subtypes (Fuzik et al., 2016; see a description on the difficulties related to the electrophysiological study of human VENs in Hodge et al., 2020). Highly multiplexed, high-resolution brain-wide cell type mapping, and high-throughput spatially resolved transcriptomics approaches would provide data to integrate individual cell type variability and connectivity-mapping information in specific brain areas (Close et al., 2021). These complementary experimental approaches can contribute with relevant data toward a unified, consensual neuronal classification based on a high-throughput single-cell transcriptomic-based taxonomy, building a probabilistic definition (Yuste et al., 2020) of spindle-shaped neurons as VENs (or not) in different brain areas and testing for intra- and inter-type variability in the phenotype of VENs at different ages. Importantly, “…the existence of cell states, spatial gradients of phenotypes and mixtures of differences and similarities in cross-species comparisons present challenges to a discrete and categorical perspective on defining cell types. Prematurely adopting an inflexible definition of types will obscure the significance of observed phenotypic variability and its biological interpretation…” while might exist a “… core and intermediate cells or the description of a cell type as a continuous trajectory in transcriptomic space” (Yuste et al., 2020).

It will be also crucial to determine if VENs emerge by differentiation of a prior cell within a cortical layer or migrate along the development toward specific parts of the cerebral mantle (Allman et al., 2005). We will have to determine if VENs exist only in cortical layer V. If so, it is interesting to question whether the spindle- to rod-shaped neuron observed in the inner layer VI of the PC (Figure 12) would resemble the shape of a layer V VEN or represents another molecularly different neuronal type in this polymorphic layer.

### Morphological Implications for Spindle-Shaped Neurons (or Putative Von Economo Neurons) in the Human Precuneus

Currently, there is no human or monkey neuron morphologically classified as VENs in the PC layer V available at the open database “NeuroMorpho.Org” (version 8.1.25, released 2021-07-22, content: 151,303 neurons), where 70 reconstructed neurons are labeled as pyramidal ones in the layer III of Brodmann parietal area 7a of the rhesus monkey, and 14 reconstructed neurons in the layer III of Brodmann parietal area 7a of the baboon and vervet monkeys (original references available at http://neuromorpho.org/KeywordResult.jsp?count=70& keywords=%22%20area%207a%22 and http://neuromorpho.org/KeywordResult.jsp?count=14&keywords=%22brodmann%20are a%207%22). Indeed, the cellular complexity, the inherent difficulties, and the technical limitations for studying the human *postmortem* brain tissue were reported previously (e.g., Dall’Oglio et al., 2013; Reberger et al., 2018; Vásquez et al., 2018 and references therein; see also the effect of the embedding medium and tissue shrinkage in the VENs spiral-shaped and corkscrew aspect of primary dendrites in Banovac et al., 2019).

The emergence of VENs is not related to the relative brain size or encephalization of the studied species (Allman et al., 2010). There is a possibility that VENs might be associated with the mechanical challenges associated with larger, gyrencephalic brains along with other evolutionary adaptations (Jacob et al., 2021). Human VENs with sparse dendritic trees and symmetric ascending and descending main shafts were considered computationally simple compared to layer V pyramidal neurons, likely receiving few inputs within individual minicolumns for a rapid cortical radial signal transmission (Watson et al., 2006). Currently, VENs with particular neurochemical profiles, heterogeneous dendritic geometry and spine features can be part of a more complex scenario than previously perceived. VENs with more branched ascending and descending dendrites (Banovac et al., 2019; Correa-Júnior et al., 2020) can have additional biophysical properties with a higher surface for synaptic processing and plasticity modulated by pleomorphic spines. Together with the intrinsic properties of putative VENs’ membrane (Hodge et al., 2020), the increased dendritic arbor might provide further possibilities for the connectivity repertoire, computational power, and elaboration of information by these cells, as described for other neuron types (Oakley et al., 2001; Wen et al., 2009; Spruston et al., 2013; Brunel et al., 2014; Eyal et al., 2016; Rollenhagen and Lübke, 2016).

Von Economo neurons express dopamine D3 and D5 receptors, serotonin-1b and −2b receptors (Watson, 2006), GABA receptor subunit θ, adrenoreceptor α-1A (Dijkstra et al., 2018), activating transcription factor 3 of the CREB protein family, interleukin-4 receptor, and neuromedin B with a possible connection of interoception/visceral states and social awareness (Allman et al., 2010; Stimpson et al., 2011; Raghanti et al., 2015). The distribution of transmitter receptors in more superficial or deeper cortical layers in the human PC (“7A of the superior parietal lobule”) suggests that ascending and descending dendrites from layer V cells may be modulated by different excitatory and inhibitory synaptic inputs (Palomero-Gallagher and Zilles, 2019). It remains to be determined whether VENs have dendritic domains with different integrative, linear and non-linear properties, and specific neurochemically modulated firing pattern as well as if VENs have heterogeneous morphological and functional features related to their intracortical or extratelencephalic projections.

Furthermore, the presence, distribution, number, size, and shape of dendritic spines from proximal to distal dendritic segments in the PC spindle-shaped neurons need to be taken into account and compared to other VENs functional properties and electrophysiology in the future. Activity-driven changes in dendritic spines can occur in a region-specific manner and according to each network and neuron-specific synaptic demand, stability, and plasticity (Benavides-Piccione et al., 2002; Chen et al., 2011; Araya et al., 2014; Hayashi-Takagi et al., 2015; Kasai et al., 2021). The spine-free dendritic zones are important for the synaptic integration (Kubota et al., 2016), but spines can provide additional properties for the modulation of neural circuitries and balancing the multimodal information processing. Spines with different forms (including those with convoluted structure) can differ in their impact on the fine-tuned synaptic processing by having different postsynaptic density composition, number and type of postsynaptic receptors, subcellular components and organization, electrical and biochemical compartmentalization, clustering pattern, degree of cooperativity between adjacent spines and the parent dendrite, and impact on the neuronal voltage and output frequency (Rochefort and Konnerth, 2012; Yadav et al., 2012; Yuste, 2013; Stewart et al., 2014; Dall’Oglio et al., 2015; Tønnesen and Nägerl, 2016; Berry and Nedivi, 2017; Lu and Zuo, 2017; Nakahata and Yasuda, 2018). Spinules, also found in PC spindle-shaped neurons, are active functional elements for synaptic development and maintenance that add to the neuronal plasticity repertoire and rapid integration of signals (Petralia et al., 2018, 2021). For example, NMDA activation can increase spinule number, length, and contact with distal presynaptic elements (Zaccard et al., 2020). There is a new avenue for research to establish the functional relation of layer V spindle-shaped neurons within the local cytoarchitecture of PC, including the activity of adjacent pyramidal and other “non-pyramidal” neurons and glia cells, in this same and in the adjacent cortical layers where ascending and descending dendrites are present.

### Possible Functional Implications for Spindle-Shaped Neurons (or Putative Von Economo Neurons) in the Precuneus Within Integrated Brain Networks

The possibility of existence of VENs in the human PC is compelling. Some interesting points deserve further discussion. That is, primates have evolved cognitive mechanisms to understand and analyze complex social interactions (Freiwald, 2020). Among the multiple cellular components with an intrinsic connectivity and functional networks in the human cerebral cortex (see further discussion in van den Heuvel et al., 2015), some large-scale circuits have been studied for their roles in resting state, attention and task-related activity or cognitive functions (Ng et al., 2016; Deming and Koenigs, 2020; Forlim et al., 2020; Kolb and Whishaw, 2021). High signal coherence within these networks makes the sub-components functionally coupled along varied timeframes (Ng et al., 2016 and references therein), such as in the DMN, which comprises the PC (Cavanna and Trimble, 2006) and the posterior cingulate cortex, the medial prefrontal cortex, and the bilateral intraparietal cortex/angular gyrus; in the “salience network” (SN), including the bilateral anterior insula and the dorsal ACC; and, in the “executive control network” (ECN) including the “frontoparietal network” (FPN) composed of bilateral middle frontal gyri and supramarginal gyri/inferior parietal lobe (Hidalgo-Lopez et al., 2021 and references therein), the intraparietal sulcus, and dorsal prefrontal cortex (Ptak, 2012; Deming and Koenigs, 2020). The PC was also included in the ECN/frontoparietal control system for moment-to-moment tasks (Kolb and Whishaw, 2021).

As a hub region of the human brain, the PMC components have been implicated in a broad array of cognitive and emotional processes (Cavanna and Trimble, 2006; Cavanna, 2007; Yang et al., 2014; Zhang et al., 2014). Multimodal information processing is reflected in the patterns of functional connectivity of brain regions. The PMC can be subdivided into clusters exhibiting connectivity profiles that are positively and negatively correlated with areas of the DMN, with a gradual transition for the PMC’s functional connectivity in the dorsal–ventral and anterior–posterior directions (Cauda et al., 2010). I.e., the dorsal–anterior parts were associated with regions subserving the control of attentional mechanisms, while the dorsal–posterior PMC, encompassing sections of Broadman areas 7 and 31, was identified as a constituent of a FPN related to visual–spatial motion control (Cauda et al., 2010). On the other hand, the ventral–anterior PMC-compartment (Broadman areas 23, 30, and 31) showed specific connections with a network strongly resemblant of the task-negative DMN, while the central-posterior PMC (containing parts of Broadman area 7) displayed links with a network related to visual information processing (Cauda et al., 2010). Employing diffusion tensor imaging and fiber tracking for the PMC, the dorsal–anterior PMC was linked up with sensorimotor areas whereas the dorsal–posterior portion was heavily tied to regions engaged in visual processing (Zhang et al., 2014). Another distinction was detected between the dorsal–central and dorsal–ventral PMC, with the former representing an associative area and the latter emerging as a transitional area between different circuitries as indicated by its highly varied set of cortical links (Zhang et al., 2014). The ventral-most section displayed extensive associations with limbic areas (Zhang et al., 2014). Taken together, these results underscore the PMC’s relevance for a multitude of interacting, yet dissociable brain networks relevant for higher-level cognition. Notably, the human PC has one of the highest resting metabolic rates in the cerebral cortex and elaborates sensorimotor, visual, and cognitive/associative information (Margulies et al., 2009), also including self-centered mental imagery and consciousness (Cavanna, 2007), empathy and perspective-taking (Zebarjadi et al., 2021), working memory (altered in cases of mild cognitive impairment, MCI, Yokosawa et al., 2020), episodic memory retrieval (Cavanna and Trimble, 2006), and metacognition processing (Ye et al., 2018).

In accordance with its involvement in cognitive functions, the human PC has been found to play a role in *g* (both structurally and functionally, see Menary et al., 2013; Basten et al., 2015; Hilger et al., 2017; Takeuchi et al., 2018). The construct of *g*, which is best conceptualized as “a distillate obtained from many diverse abilities” (Jensen, 1998), is regarded as the single best predictor of scholastic and vocational achievements as well as other socially relevant outcomes (Gottfredson, 1997; Jensen, 1998). Due to its association with neurological variables (Penke et al., 2012; Gignac and Bates, 2017), *g* has become a major focus of investigation (Haier, 2016), and the influential “Parieto-Frontal Integration Theory” (Jung and Haier, 2007) posits that *g* is closely related to a task-invariant network comprising a circumscribed set of brain regions. Several lines of evidence support the existence of such a domain-general network (Basten et al., 2015; Hugdahl et al., 2015). Regions often implicated in the latter include prefrontal areas such as the dorsolateral prefrontal cortex, the ACC and anterior insula, as well as parietal areas such as the intraparietal sulcus and PC (Jung and Haier, 2007; Niendam et al., 2012; Basten et al., 2015; Assem et al., 2020; Martínez and Colom, 2021). As its prefrontal regions exhibit an intriguing overlap with those ones to which human VENs are restricted, the existence of VENs in the parietal portion of the “process-invariant network” associated with *g* has been predicted (Bruton, 2021). The plausibility of this prediction, suggested by the present findings, is underscored by the fact that the PMC has been shown to play a role in two intrinsic connectivity networks (ICNs) known to substantially relate to *g*: namely, the central executive network (CEN) and the DMN (Cauda et al., 2010; Utevsky et al., 2014; Dubois et al., 2018; Uddin et al., 2019). The ability to activate the CEN while efficiently deactivating the DMN has been found to predict higher levels of *g* and abilities known to be highly saturated with *g*, such as working memory (Anticevic et al., 2012; Basten et al., 2013; Gignac, 2014; Koshino et al., 2014; Sherman et al., 2014; Bruton, 2021; DeSerisy et al., 2021). Thus, the DMN’s relevance for *g* may stem from the fact that the insufficient and inefficient attenuation of this network may interfere with the successful employment of externally oriented, task-related attentional resources – a task accomplished by the ECN (Anticevic et al., 2012; Basten et al., 2013; Hugdahl et al., 2015; Bruton, 2021).

In this context, Bruton (2021) elaborated another possibility regarding the functional role of human VENs integrated in brain circuits. It was hypothesized that VENs might contribute to the emergence of *g* by functioning as cerebral pacemakers that promptly establish the coherence of neuronal oscillations (Bruton, 2021). This conjecture is grounded in the “communication-through-coherence” hypothesis which contends that the interaction between connected neuronal populations is limited to temporal slots of synchronized oscillations (Fries, 2005, 2015). VENs would thus transmit rhythmic signal bursts across large distances that, after arriving at their targeted neuronal populations, entrain the latter to the signaled rhythm, thereby implementing oscillatory coherence. In this manner, VENs might function as so-called “herald neurons” which allow for the undelayed processing of subsequently arriving, more complex signals (Bruton, 2021). This conceptualization might also help to elucidate the VENs presumed role in switching between the anticorrelated ECN and DMN (Menon and Uddin, 2010; Bruton, 2021). These VENs would facilitate the unobstructed communication between critical frontal and parietal regions and subdivisions implicated in the ECN and DMN, respectively (Bruton, 2021). Instead of directly activating the ECN while disabling the DMN (Sridharan et al., 2008), VENs (as “herald neurons”) would rapidly establish coherence of oscillations between core regions of both networks prior to the phase of actual switching, leading to more efficient functional dissociation and thus decreased DMN interference with task-relevant mental activity originating from the ECN (Anticevic et al., 2012; Bruton, 2021). The fact that different subdivisions of the PMC, including the PC, have been implicated in different ICNs (Margulies et al., 2009; Yang et al., 2014; Kolb and Whishaw, 2021) underlines the importance of efficient internetwork communication especially in this particular parietal area to which VENs could significantly participate in (for additional data on PMC and PC connectome, tractography and white matter dissection, see Baker et al., 2018; Assem et al., 2020; Jitsuishi and Yamaguchi, 2021). An intriguing objective for future research would be to establish whether the observed variation in dendritic architecture and spine features of spindle-shaped neurons, if they are VENs, exhibit individual differences and whether they are correlated with human cognition as reflected by constructs such as *g*.

### Vulnerability of Von Economo Neurons and the Pathological Findings Involving the Human Precuneus

The PC is also directly or indirectly affected by neuropsychiatric disorders in which VENs would show vulnerability or pathological involvement (Nimchinsky et al., 1995; Allman et al., 2001, 2005; Watson, 2006; Cauda et al., 2014; Raghanti et al., 2015; Jacot-Descombes et al., 2020). Putative VENs are affected in the behavioral variant of the frontotemporal dementia with hindered social–emotional functions (Seeley et al., 2006; Seeley, 2008; Santillo and Englund, 2014; Gami-Patel et al., 2019; Lin et al., 2019); in cases of agenesis of the corpus callosum with impairment in humor and judgment of scenes of social interactions; in the bipolar disorder (reviewed in Cauda et al., 2014; Raghanti et al., 2015) and autism spectrum disorder (Allman et al., 2005), and in familial dysautonomia with mood impairments (Jacot-Descombes et al., 2020).

Von Economo neurons were also related to schizophrenia (Krause et al., 2017). The protein encoded by the gene DISC1 (disrupted in schizophrenia) had an evolutionary change in the line leading to humans, is related to neuronal migration and dendritic branching, and is preferentially expressed by VENs (Allman et al., 2010; Cauda et al., 2014; see the GWAS atlas at https://atlas.ctglab.nl for current data on current DISC1 associations). In addition, reduced resting-state connectivity in the PC was correlated with apathy in patients with schizophrenia (Forlim et al., 2020). Apathy is a motivational disorder incurring reduced or loss of goal-directed behavior, goal-directed cognitive processes, and emotion (Forlim et al., 2020). In this regard, the PC connectivity was correlated with the severity of these symptoms and with alterations in the subjective experience of a continuous sense of the self, regarded as dysfunctional interactions between relevant brain regions (Forlim et al., 2020). On the other hand, in few cases when epilepsy originated in the PC there were heterogeneous ictal symptoms, including body movement sensation or body image disturbance, somatosensory and visual auras, vestibular manifestations, eye movements, complex motor behaviors, and bilateral asymmetric tonic and hypermotor seizures (Harroud et al., 2017; Yang et al., 2018).

A meta-analysis of functional MRI task-related activity of psychopathy drew attention to the increased activity predominantly in midline cortical regions overlapping with the DMN (i.e., dorsomedial prefrontal cortex, posterior cingulate, and PC) as well as with the medial temporal lobe (Deming and Koenigs, 2020). Psychopathy was negatively related to neural activity in dorsal ACC and was positively related to neural activity in a bilateral portion of medial parietal and occipital cortex (including posterior cingulate and PC), bilateral dorsomedial prefrontal cortex, right inferior frontal gyrus, right posterior orbitofrontal cortex, right medial temporal cortex (including amygdala), right hippocampus, and right parahippocampal gyrus (Deming and Koenigs, 2020). Whereas DMN increases activity during self-referential processing and decreases activity during externally focused, non-self-referential tasks, FPN increases activity during cognitively demanding, externally focused tasks (Deming and Koenigs, 2020). The SN, which is particularly important for detecting salient external stimuli, would be responsible for switching between the two anticorrelated networks, DMN and FPN (Deming and Koenigs, 2020). In psychopathy, both the DMN components posterior cingulate cortex and PC not only are overactive across a variety of tasks but also less functionally and structurally connected to other DMN regions, including dorsomedial and ventromedial prefrontal cortex and other regions of the FPN (Deming and Koenigs, 2020). One possibility is that psychopathic individuals fail to deactivate midline DMN regions during externally focused tasks and such failure could result in increased competition between DMN and externally oriented attention networks (such as FPN), disrupting the shift of attention to the external task, and leading to corresponding performance deficits (Deming and Koenigs, 2020).

The PC neurons and circuitries can also be damaged in Alzheimer’s disease (AD; Gefen et al., 2018; Berron et al., 2020; but see also Nelson et al., 2009). Metabolic reduction or hypoperfusion of the PC can be found in the early stages of AD, even before clinical diagnosis of dementia (Thomas et al., 2019). It is possible that hypoperfusion begins largely in PC and spreads to the parietal cortex and cingulate gyrus along with progression of the AD to other cortical areas (Aghakhanyan et al., 2018). The PC is involved in working memory and its deactivation is associated with early MCI assessed by a validated clinical screening instrument for cognitive decline (Yokosawa et al., 2020). Moreover, the PC seems to be a region with high vulnerability to β-amyloid (Aβ) deposition, showing major Aβ load in Aβ-PET and in association with cognitive decline in MCI and AD patients compared with healthy controls (Aghakhanyan et al., 2018). White matter microstructure alteration occurs in the early stage of amyloid pathology and the strongest association was found in PC and the corpus callosum (Collij et al., 2021). The study of the cellular components of the PC and the timely diagnosis of an altered function in this brain area would direct new treatments to alleviate or prevent the progress of MCI and AD in the future.

In conclusion, we describe the presence of Nissl-stained spindle-shaped neurons in the anterior and central regions of the human PC with a cell body aspect in layer V compatible with putative VENs, as described in other brain areas. The Golgi morphological identity of these cells has now to be complemented with relevant neurochemical, electrophysiological, and transcriptomic-based identification for unified cell type classification. Using the Golgi method, the PC spindle-shaped neurons in layer V and in the transition to layer VI have a dendritic architecture and spine diversity that suggest additional functional implications for the local cytoarchitecture and for the synaptic and information processing in integrated networks for higher-order activities (such as the DMN and *g*) in this multimodal complex area. Additional studies can elucidate the transcriptomic features of the PC spindle-shaped (or putative VENs) and compare them with other cells in the human ACC and FI, considering network circuits demands with particular local features and/or diversity in the morphological and functional cellular specializations in the human brain. This will be an important achievement also for the comprehension of neuropsychiatric disorders involving the PC in the context of the PMC functioning.

## Data Availability Statement

The datasets presented in this study can be found in online repositories. The names of the repository/repositories and accession number(s) can be found below: Raw data included in this manuscript were generated at the UFCSPA (Brazil) and are available from the authors. Data were originally presented and registered as a M.Sc. Thesis (2018) as follows: Fuentealba Villarroel, F.J. “Estudo sobre os neurônios de von Economo do pré-cúneo humano” (in Portuguese), Graduation in Neurosciences, Universidade Federal do Rio Grande do Sul (UFRGS), Brazil, publicly registered at https://www.lume.ufrgs.br/handle/10183/193616.

## Ethics Statement

The studies involving human participants were reviewed and approved by the Brazilian Ethics Committee from the Federal University of Health Sciences of Porto Alegre (UFCSPA; #62336116.6.0000.5345, 18718719.7.0000.5345, and 06273619.7.0000.5345) and the Federal University of Rio Grande do Sul (#18718719.7.3001.5347). The next of kin of the patients/participants provided their written informed consent for brain donation during autopsy.

## Author Contributions

FJF-V, JR, AH, and AAR-F: study concept and design. FJF-V, JR, and AAR-F: acquisition of data and two-dimensional reconstructions. JR and AAR-F: three-dimensional reconstructions. FJF-V, JR, AH, OJB, and AAR-F: critical interpretation of data and discussion and elaboration of the manuscript. All authors contributed to the article and approved the submitted version.

## Conflict of Interest

The authors declare that the research was conducted in the absence of any commercial or financial relationships that could be construed as a potential conflict of interest.

## Publisher’s Note

All claims expressed in this article are solely those of the authors and do not necessarily represent those of their affiliated organizations, or those of the publisher, the editors and the reviewers. Any product that may be evaluated in this article, or claim that may be made by its manufacturer, is not guaranteed or endorsed by the publisher.

## Footnotes

1
https://imagej.nih.gov/ij/plugins/volumeviewer.html

## Supplementary Material

Supplementary Figure 1 Three-dimensional reconstruction of layer V Golgi-impregnated from the human precuneus cortex (corresponding to Figure 5).

Supplementary Figure 2 Three-dimensional reconstruction of layer V Golgi-impregnated from the human precuneus cortex (corresponding to Figure 6).

Supplementary Figure 3 Three-dimensional reconstruction of layer V and in the transition to layer VI Golgi-impregnated from the human precuneus cortex (corresponding to Figure 7).

Supplementary Figure 4 Golgi-impregnated layer V pyramidal neuron from the human precuneus cortex (central region, 61.1 mm posterior to the midpoint of the anterior commissure).
