# Supplementary figures and images for: Spindle-Shaped Neurons in the Human Posteromedial (Precuneus) Cortex
Source: Front Synaptic Neurosci. 2022 Jan 11;13:769228. doi: 10.3389/fnsyn.2021.769228 (PMC8787311; doi:10.3389/fnsyn.2021.769228)

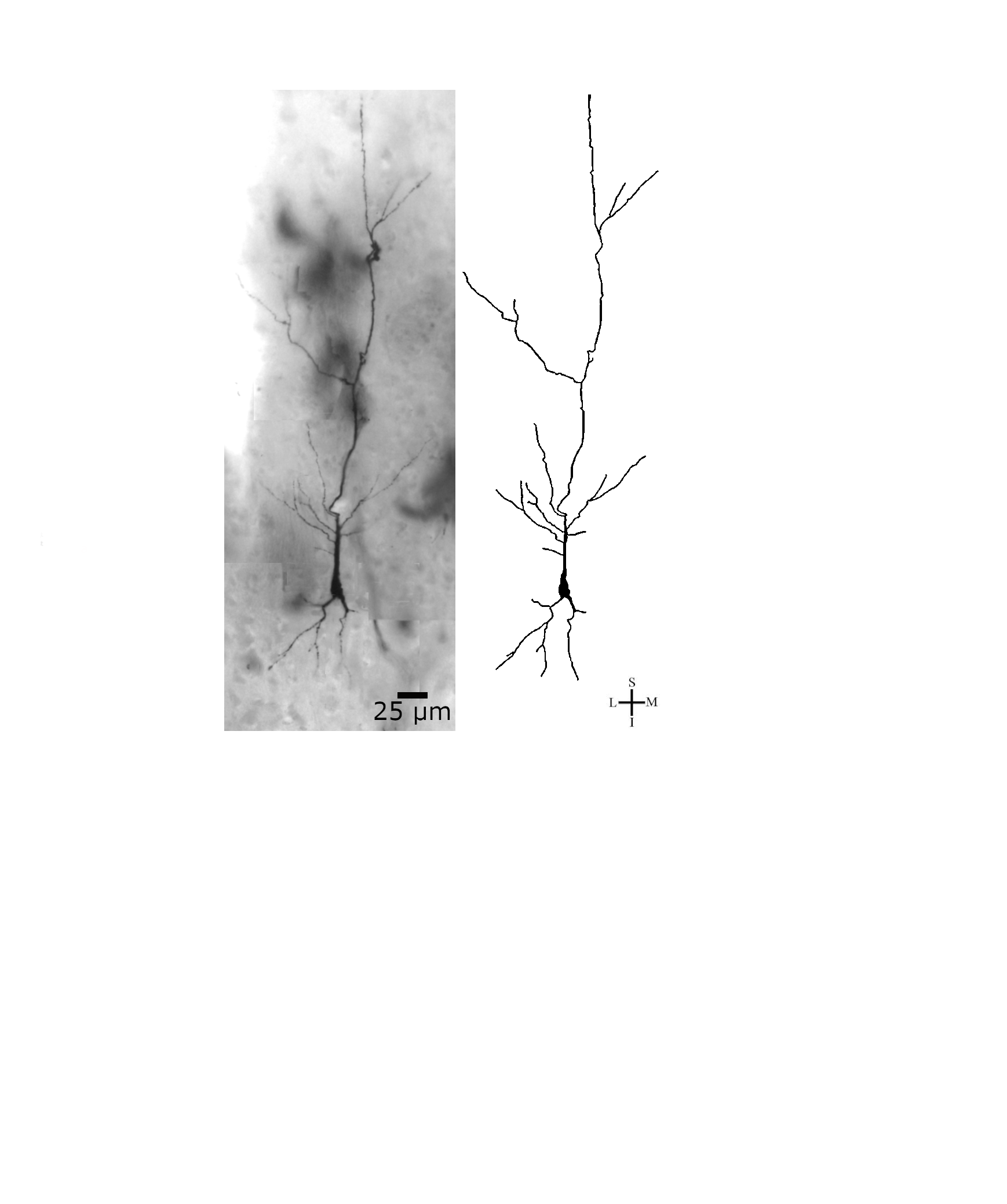

Supplement: Supplementary Figure 4 — Golgi-impregnated layer V pyramidal neuron from the human precuneus cortex (central region, 61.1 mm posterior to the midpoint of the anterior commissure). [file Image_4.tif]
